# Dermoscopy of External Ear Melanocytic Lesions: Performance of Selected Dermoscopic Screening Algorithms and Proposal of a New Predictive Model for Malignancy (AuriCheck Dermoscopic Algorithm)

**DOI:** 10.3390/cancers17040679

**Published:** 2025-02-17

**Authors:** Jakub Żółkiewicz, Luc Thomas, Grażyna Kamińska-Winciorek, Krzysztof Pastuszak, Michał Kunc, Urszula Maińska, Michał Sobjanek, Martyna Sławińska

**Affiliations:** 1 Department of Dermatology, Venereology and Allergology, Medical University of Gdansk, 80-214 Gdansk, Poland; 2 Department of Dermatology, Venereology and Allergology, University Clinical Center in Gdansk, 80-211 Gdansk, Poland; 3 Department of Dermatology, Centre Hospitalier Lyon Sud, 69495 Lyon, France; 4 Lyon 1 University, 69100 Lyon, France; 5 Lyons Cancer Research Center UMR INSERM U1052-CNRS5286-UCBL1, 69008 Lyon, France; 6 Skin Cancer and Melanoma Team, Department of Bone Marrow Transplantation and Onco-Hematology, Maria Sklodowska-Curie National Research Institute of Oncology, Gliwice Branch, 44-102 Gliwice, Poland; 7 All4Skin, Center of Diagnostics and Therapy of Skin Diseases, 40-750 Katowice, Poland; 8 Department of Algorithms and System Modelling, Gdansk University of Technology, 80-214 Gdansk, Poland; 9 Laboratory of Translational Oncology, Intercollegiate Faculty of Biotechnology, University of Gdansk and Medical University of Gdansk, 80-214 Gdansk, Poland; 10 Center of Biostatistics and Bioinformatics, Medical University of Gdansk, 80-214 Gdansk, Poland; 11 Department of Pathomorphology, Medical University of Gdansk, 80-214 Gdansk, Poland

**Keywords:** dermoscopy, external ear, melanoma, nevus, algorithm, diagnostics

## Abstract

**Background/Objectives:** External ear melanocytic lesions (EEMLs) are frequently encountered in clinical practice. EEMLs belong to dermoscopic special site locations and their dermoscopic presentation is poorly characterized. The aims of the study were to determine the dermoscopic features and patterns of EEMLs, to evaluate the performance of selected dermoscopic screening algorithms for EEML and to propose a dedicated site-specific dermoscopic predictive model for the assessment of EEMLs. **Methods:** A retrospective study of EEMLs, which were either excised or had ≥2 years of stable clinical and digital dermoscopy follow-up, was performed. Each lesion was evaluated according to the predefined criteria and selected dermoscopic screening algorithms. Based on the diagnostic potential of each dermoscopic feature, the predictive model was developed and validated utilizing a test set derived from samples obtained from the HAM10000 database. **Results:** A total of 145 lesions from 136 patients (63 females; 46.3%) were analysed. The mean age of patients was 45.6 years. The most frequent dermoscopic findings of malignant lesions were irregular pigmentation (50/56; 89.3%) and asymmetry of the pattern (49/56; 87.5%), followed by asymmetry of colour (48/56; 85.7%), whereas the most common dermoscopic features of benign lesions were the presence of brown structureless areas (69/89; 77.5%), point and axial symmetry of pigmentation (63/89; 70.8%) and the presence of dots/globules (45/89; 50.6%). Among the selected dermoscopic screening algorithms, the 7-point checklist reached the highest sensitivity of 92.9%, whereas the CASH algorithm achieved the highest specificity of 93.3%. The predictive model reached a balanced accuracy of 88.3% on the independent test set and 89.5% on the validation set. **Conclusions:** This is the first large-scale study on the dermoscopic features of EEMLs. A predictive diagnostic model was proposed to aid external ear melanoma recognition in the clinical setting. Previously unreported dermoscopic feature (‘red circles’) and dermoscopic pattern (‘annular-globular pattern’) were described.

## Simple Summary

The data on dermoscopic presentation of external ear melanocytic lesions (EEMLs) are limited. We aimed to summarize the dermoscopic features of EEMLs, assess the performance of selected dermoscopic screening algorithms and propose a dermoscopic predictive model dedicated to this location. Previously undescribed dermoscopic structure (red circles) and pattern (annular-globular) were identified. The proposed predictive dermoscopic model has demonstrated both high sensitivity and specificity (hovering around 90%) for the assessment of EEMLs. This is the first large-scale study on the dermoscopic features of EEMLs. The proposed dermoscopic model was superior compared to known dermoscopic screening algorithms. The description of novel dermoscopic structure, pattern and the predictive model may aid early external ear melanoma diagnosis.

## 1. Introduction

Dermoscopy is a non-invasive imaging technique that significantly enhances diagnostic accuracy in diagnosing skin disorders [1]. The diversity of skin anatomy implies specific dermoscopic features associated with different skin locations. Hence, the term ‘special site location’ (SSL) dermoscopy has been coined to highlight the distinctiveness of dermoscopic patterns depending on a lesion’s location [2,3]. The dermoscopic patterns of melanocytic lesions observed on glabrous skin have been extensively studied; however, their significance in the assessment of SSL lesions is underinvestigated. In recent years, dedicated site-specific algorithms for the differential diagnosis of facial [4,5] and nail unit lesions [6,7] have been validated and successfully applied in the clinical setting.

External ear melanocytic lesions (EEMLs) belong to dermoscopic SSL. Rishpon et al. [8] revealed that among 564 consecutive patients undergoing total body examination in the pigmented lesion clinic, almost 30% presented with at least one pigmented skin lesion located on the external ear. Despite the fact that they are frequently encountered in clinical practice, EEMLs remain a remarkable diagnostic challenge for physicians.

External ear melanomas (EEMs) are characterized by worse outcomes compared to other anatomical locations [9,10]. Improved recognition of EEMs aided by dermoscopy may enhance early identification and treatment, ultimately improving outcomes. Nevertheless, data on dermoscopic patterns of melanocytic lesions on the external ear, including EEMs, remain limited, with only a few studies published to date, each covering a small number of cases [11,12,13,14].

The aim of this study was to identify dermoscopic features and patterns of EEMLs. The secondary aims were to validate selected dermoscopic screening algorithms for EEMLs and to propose a dermoscopic model dedicated for assessment of EEMLs.

## 2. Materials and Methods

A retrospective, cross-sectional, observational study was designed to evaluate the dermoscopic features of melanocytic skin lesions located on the external ear. The study was conducted across four dermatology centres located in Gdańsk, Gliwice, Katowice (Poland) and Lyon (France), and included patients who were consulted between January 2008 and December 2023. All histopathologically confirmed lesions and non-excised lesions of the ear with at least 2 years of clinical and videodermoscopic follow-up period were included in the analysis. The diagnosis of excised lesions relied on histopathological examinations performed by the dermatopathologist specialized in the diagnosis of skin neoplasms at each participating institution. Non-melanocytic pigmented lesions (e.g., seborrheic keratosis, pigmented basal cell carcinoma), lesions exceeding the size of the imaging area (preventing full visualization of the lesion’s surface) and non-excised lesions with follow-up periods shorter than 2 years were excluded from the analysis.

Clinical data for each patient were collected (as listed in Table 1), encompassing age, gender, skin phototype, lesion diameter, site and specific location within the external ear. Additionally, for histopathologically confirmed cases, information on whether the lesion was removed immediately after detection or after a follow-up period was collected (in the latter case, duration of the observation period was also recorded). For non-excised lesions, the duration of the follow-up period was documented. Both for excised lesions and cases that were followed-up with digital dermoscopy, the baseline digital dermoscopy image was analysed. All excised lesions were assigned to either the benign or malignant group based on the detailed histopathological diagnosis. Atypical Spitz tumours, Superficial Atypical Melanocytic Proliferations of Unknown Significance (SAMPUS), Melanocytic Tumours of Uncertain Malignant Potential (MELTUMP) and Intraepidermal Atypical Melanocytic Proliferation of Uncertain Significance (IAMPUS) were included in the malignant category in the current study as their diagnosis requires further medical management and surveillance. In melanoma cases, Breslow thickness was retrieved.

### 2.1. Dermoscopy Protocol

Details regarding the dermoscope models used, dermoscopic examination techniques, and the image analysis protocol are provided in Supplementary Text S1.

**Figure 1:**
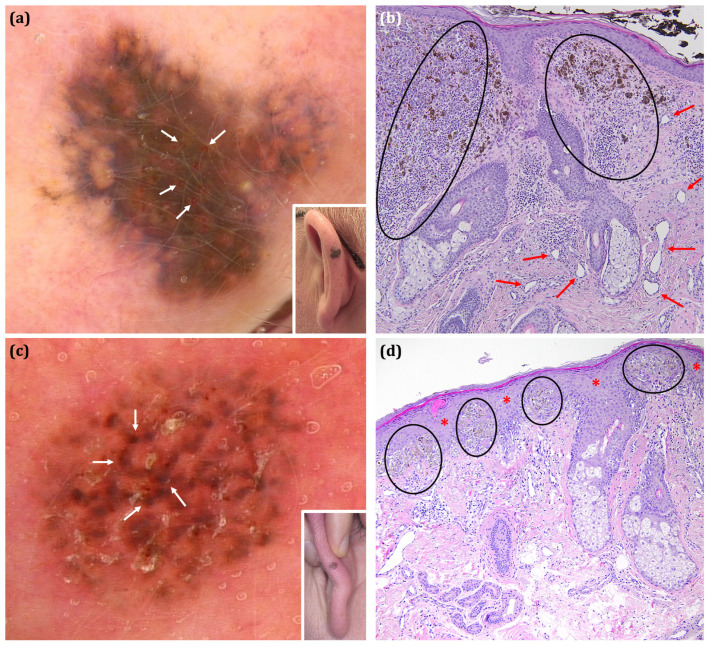
The clinical, dermoscopic and histopathological presentation of the previously undescribed structure and pattern—‘red circles’ and ‘annular-globular pattern’. (**a**,**b**) **‘The red circles’ in nevoid melanoma.** (**a**) The red circles are defined as the presence of continuous, complete red circles located at the periphery of the follicular openings (white arrows). (**b**) Round nests of atypical melanocytes with impaired maturation in depth (black ellipses) are seen in the corresponding histopathological image. Dilated perifollicular vessels are prominent (red arrows), what may correspond to the presence of ‘red circles’ observed in the dermoscopy. (**c**,**d**) **The annular-globular pattern’ in Atypical Spitz Tumour.** (**c**) The ‘annular-globular pattern’ represents globules (instead of granules) arranged concentrically around hair follicle ostia (white arrows). According to our observations, it may be characteristic for the auricular area; however, this is probably not helpful in differentiating between benign and malignant EEMLs. (**d**) Large diameter nests located in the interfollicular spaces (black ellipses) observed in the histopathological examination may correspond to the brown globules constituting the annular-globular pattern. Melanocytes within the nests are visible in the interfollicular spaces (hair follicles are marked with red asterisks). (**a**,**c**) FotoFinder, Medicam 800HD [FotoFinder Systems GmbH, Bad Birnbach, Germany]; 20× magnification, immersion gel. (**b**,**d**) Hematoxylin and eosin staining; magnification, 40×.

Dermoscopic analysis included the following parameters: colours, vascular structures, dermoscopic features specific for facial melanoma and dermoscopic features specific for melanoma in general (hereinafter also called ‘facial’ and ‘extra-facial’ melanoma dermoscopic features). Variables were selected based on the third International Dermoscopy Society consensus document [15]. As no large size study and dermoscopic terminology on external ear melanoma has been published to date, additional dermoscopic features were adopted from previous studies on melanocytic lesions located on the external ear [8,12,16]. Moreover, based on our preliminary observations prior to the conducted analysis, additional feature—‘red circles’—and pattern—‘annular-globular pattern’—were also evaluated. Since the ‘red circles’ and ‘annular-globular pattern’ have not been previously described, we propose the following definitions of these novel findings: ‘red circles’: “the presence of a continuous, complete red circle located at the periphery of the follicular opening”; ‘annular-globular pattern’: “the presence of globules arranged around follicular openings”. While the new ‘annular-globular pattern’ resembles the melanoma-associated ‘annular-granular pattern’, it differs from the latter by the presence of globules instead of granules. The new dermoscopic structure and pattern along with the corresponding histopathological images are presented in Figure 1.

Moreover, a pattern consisting of “pigmented circles with thick line(s) originating from them” was evaluated. The morphological aspect of these structures and their potential diagnostic significance have been analysed in the context of the previously described “perifollicular linear projections” by Navarrete-Dechent et al. [17]. To objectively evaluate whether EEMLs dermoscopically resemble facial or extra-facial pigmented lesions, all cases were categorized into either ‘facial’ or ‘extra-facial’ phenotypes. A lesion was classified as having a ‘facial’ phenotype when it exhibited more dermoscopic structures characteristic for facial melanoma than those typical for extra-facial melanoma (as listed in Table 2), with a pseudonetwork also considered a feature of the facial phenotype. Additionally, each lesion was evaluated according to the following dermoscopic screening algorithms: Seven Non-melanoma Features to Rule Out Facial Melanoma, 7-point Checklist, ‘Chaos and Clues’, CASH algorithm, Menzies method and 3-point Checklist [18,19,20,21,22,23]. All variables of the respective algorithms were also assessed. The overall accuracy of each algorithm was calculated and served as a reference point for assessing the sensitivity and specificity of the newly designed predictive model for melanocytic lesions located on the external ear. A detailed list of all analysed variables is presented in Table 2.

### 2.2. Statistical Analysis and Model Development

The data on statistical methods are provided in Supplementary Text S1.

The algorithm development process followed a structured, three-stage approach to ensure robust and clinically relevant outcomes. Initially, the data were divided into training and validation sets, with the HAM10000 cohort designated as an independent test dataset.

Data partitioning into training and validation sets was performed in a stratified manner using a 70:30 ratio. Although the hospital where the samples were collected was not included in the stratification process, the distribution of patients from the same hospital between the training and validation sets approximately followed the 70:30 ratio. Next, a set of candidate dermoscopic features was identified by analyzing their positive and negative predictive values within the training set. Models were trained using the five or six most important features, and their performance was evaluated on the validation set. Receiver operating characteristic (ROC) curve analysis and the Youden index guided the selection of an optimal decision threshold for each model. Feature sets from the best-performing models were then reviewed by experienced dermatologists to confirm their clinical relevance and applicability.

A test set of dermoscopic images of external ear melanocytic lesions was obtained from the HAM10000 dataset to validate the proposed dermoscopic predictive model [24]. Suitable lesions were identified by applying an ‘ear’ localization filter. Melanocytic lesions (melanoma and melanocytic nevi) confirmed through histopathology were included in the further analysis and ultimately constituted the test set for the proposed model. Cases diagnosed based on follow-up examinations or expert consensus and any entities other than melanocytic ones identified in the repository were not included.

Subsequently, each case from the test set was evaluated according to the methodology described in the ‘Dermoscopic analysis’ section. Moreover, age and sex were retrieved for each case as they were the only demographic features available in this public dataset. A total of 25 histopathologically confirmed auricular melanocytic lesions were extracted from the HAM10000 dataset. The dataset search returned 10 cases of melanoma and 15 cases of nevi. The test set consisted of 13 female cases and 12 male cases, with a mean age of 57.4 years (age data were missing for two patients). Compared with our main cohort, patients from the HAM10000 dataset were older (median age of 60 vs. 43 years, *p* = 0.00905), whereas sex distribution did not differ significantly (52% female in HAM10000 vs. 46.2% in our cohort, *p* = 0.6668).

Ultimately, the selected model hyperparameters were determined using a grid search, resulting in the selection of gamma = 0, eta = 0.3, subsample = 0.7 and colsample = 0.7. Finally, the model’s performance was tested on the independent set, utilizing the decision threshold established during the validation phase.

## 3. Results

### 3.1. Clinical Assessment

**Figure 2:**
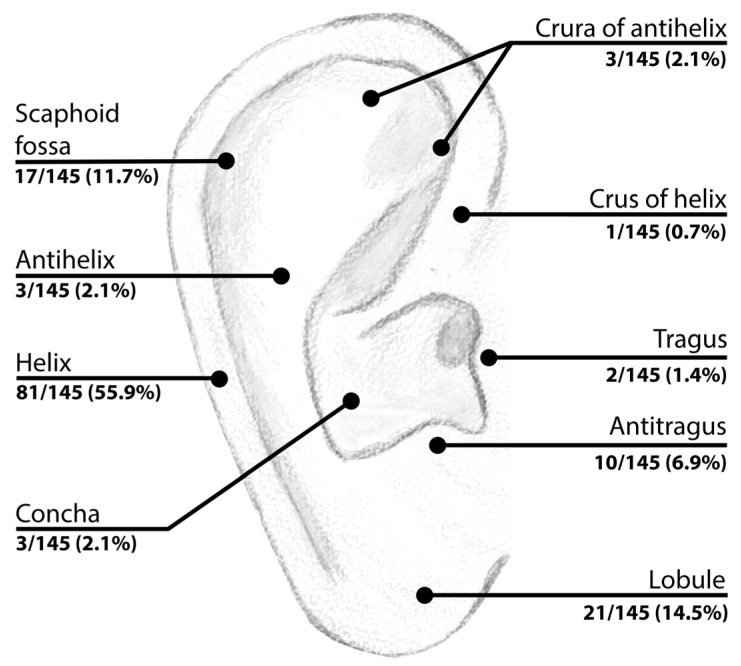
Distribution of the studied lesions across external ear anatomical sublocations. A total of 4 out of 145 (4/145; 2.8%) lesions were located within the posterior part of the auricle.

A total of 145 lesions from 136 patients (63 females; 46.3%) were included in the final analysis. The mean age of patients was 45.6 years (median age 46.5 years; range 4–88 years). The Fitzpatrick’s skin phototypes of included patients were as follows: I-33, II-82, III-21, IV-0, V-0 and VI-0. Of all analysed lesions, 105 were excised (*n* = 105/145; 72.4%), and the remaining 40 (*n* = 40/145; 27.6%) were classified as benign based on dermoscopic follow-up (≥24 months). The majority of histopathologically verified lesions were excised immediately after detection (90/105; 85.7%). The mean follow-up period of EEMLs that later underwent diagnostic excision (15/105; 14.3%) was 24.9 ± 24.1 months, whereas corresponding follow-up period for non-excised lesions was 45 ± 31.9 months. Among the lesions evaluated histopathologically, 56 were diagnosed as malignant (*n* = 56/105; 53.3%) and 49 as benign melanocytic lesions (49/105; 46.7%). Patients in the malignant group were significantly older than those in benign group (mean age of 58.3 vs. 37.7 years, *p* < 0.0001). Out of 48 melanomas, 30 were diagnosed in males and 18 in females (30/48; 62.5% vs. 18/48; 37.5%, *p* = 0.1114; male-to female ratio of 1.67:1) (Table 1). The distribution of lesions across anatomical locations is illustrated in Figure 2.

### 3.2. Dermoscopic Analysis

The most frequent dermoscopic features of malignant lesions were irregular pigmentation (50/56; 89.3%) and asymmetry of pattern (49/56; 87.5%), followed by asymmetry of colour (48/56; 85.7%), whereas the most common dermoscopic features of benign lesions were the presence of brown structureless areas (69/89; 77.5%), point and axial symmetry of pigmentation (63/89; 70.8%) and the presence of dots/globules (45/89; 50.6%) (Table 2).

Among the most impactful general melanoma features were the presence of polymorphic vessels and asymmetry of colour and structure. The mean number of colours seen under dermoscopy in malignant lesions was higher than in benign lesions (4.1 vs. 2.1; *p* < 0.0001). The novel dermoscopic feature of ‘red circles’ was identified in a limited number of cases; however, it demonstrated relatively high specificity for malignancy. ‘Red circles’ were identified in 6 out of 56 malignant lesions and in only 1 case of Spitz nevus (6/56; 10.7% vs. 1/89; 1.1%, *p* = 0.0135). The ‘annular-globular pattern’ was identified in 11 out of 145 lesions (11/145; 7.6%), of which 4 were malignant and 7 were benign (4/56; 7.1% vs. 7/89; 7.9%, *p* = 1).

An annular-granular pattern, asymmetric pigmented follicular openings, hyperpigmented follicular openings, increased density of vascular network (defined as a vascular network of higher density within the lesion than in peripheral skin), polygons/zig-zags, red rhomboidal structures and rhomboids were ‘facial melanoma’ dermoscopic features strongly associated with malignancy (*p* values of all enumerated features were <0.0001). A circle within a circle, along with dark blotches and obliterated hair follicles, were other ‘facial melanoma’ dermoscopic features highly indicative of malignancy (*p* values of both features <0.001).

Key ‘extra-facial’ dermoscopic features supportive for malignancy were irregular pigmentation, regression (manifesting as erythema/vessels/scar-like depigmentation) and milky-red areas (*p* values <0.0001). Irregular globules, negative network, blue-white veil and regression (manifesting as granularity/peppering) were other significant ‘extra-facial’ dermoscopic features associated with malignancy (*p* values < 0.006).

The majority of analysed lesions (72/145; 49.7%) were assigned to the ‘facial’ dermoscopic phenotype. Forty-two lesions demonstrated an extra-facial phenotype and the remaining 31 cases had an equivalent number of respective features (42/145; 29% and 31/145; 21.4%, respectively).

**Figure 3:**
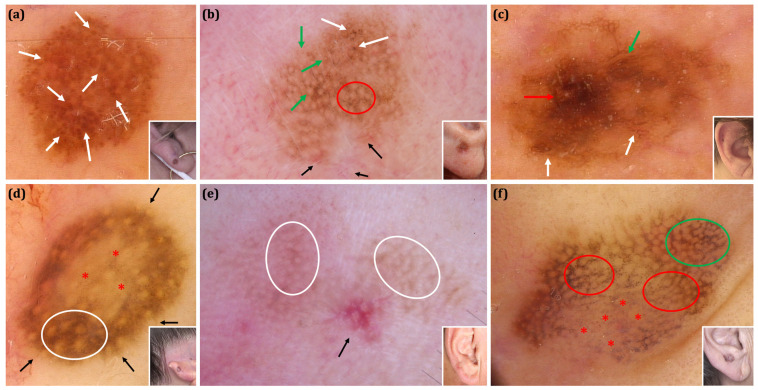
The clinical and dermoscopic spectrum of malignant EEMLs. (**a**) Superficial spreading melanoma (Breslow thickness 0.5 mm) of the right lobule (inset). The predominant dermoscopic structures observed on dermoscopy are regularly distributed brown globules (white arrows). The lesion is relatively symmetric in both pattern and colour and was identified as suspicious in only one of the analysed algorithms—’7-non melanoma features to rule out melanoma’. (**b**) Lentigo maligna of the right lobule (inset). Dermoscopy shows asymmetric pigmented follicular openings (green arrows) along with focal areas with an annular-granular pattern (red ellipse). Increased density of vascular network (black arrows) and irregular globules (white arrows) are also observed. (**c**) Lentigo maligna of the right scaphoid fossa (inset). Mixed pattern comprising atypical pigmented network (white arrows), parallel lines/fingerprinting (green arrow) and a brown structureless area (red arrow) is observed on dermoscopy. (**d**) Superficial spreading melanoma (Breslow thickness 0.3 mm) of the left helix (inset). A central area of regression manifesting as granularity/peppering (red asterisks) surrounded by a pseudonetwork (white ellipse) is visible on dermoscopy. Moreover, segmental radial lines are seen on the periphery of the lesion (black arrows). (**e**) Lentigo maligna melanoma (Breslow thickness 0.5 mm) of the right lobule (inset). Dermoscopy reveals the presence of a pseudonetwork (white ellipses). A small cluster of red rhomboidal structures is shown in the central bottom part of the picture (black arrow). The image was acquired in a polarized mode, and rosettes are therefore visible over red rhomboidal structures. (**f**) Superficial Atypical Melanocytic Proliferations of Uncertain Significance (SAMPUS) of the right lobule (inset). Dermoscopy shows an annular-granular pattern (red ellipses) associated with regression features [peppering and milky-red areas - (red asterisks)]. Focally, asymmetric pigmented follicular openings, a pseudonetwork and polygonal structures are observed (green ellipse). (**a**,**c**,**d**,**f**) FotoFinder, Medicam 800HD [FotoFinder Systems GmbH, Bad Birnbach, Germany]; 20× magnification, immersion gel. (**b**,**e**) DermLite Foto dermoscope [3Gen, San Juan Capistrano, USA] attached to a Nikon Coolpix 995 digital camera [Nikon, Tokyo, Japan]; 10× magnification, polarized light.

**Figure 4:**
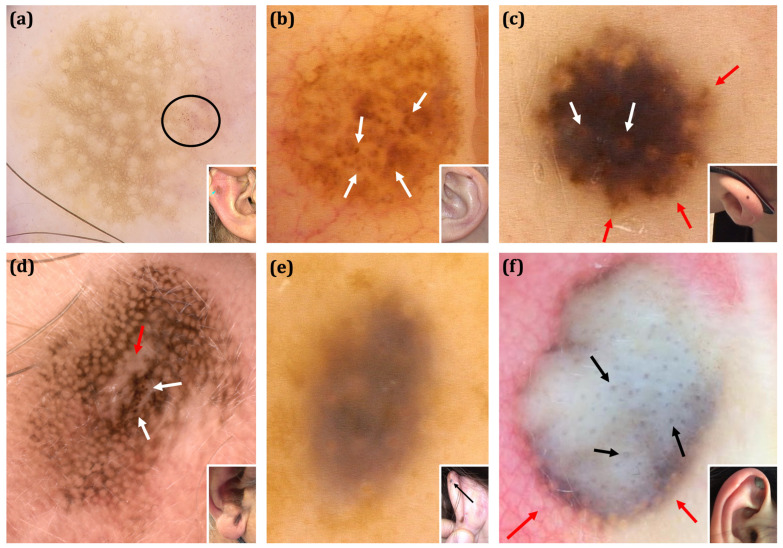
The clinical and dermoscopic spectrum of benign EEMLs. (**a**) Benign nevus of scaphoid fossa of the right ear (inset) considered benign based on over 3 years of stable clinical and videodermoscopic observation. Dermoscopy shows a typical pigmented network pattern with an eccentric area containing dotted vessels located within the ”holes” of the pigmented network (black ellipse). (**b**) Benign nevus of the left helix considered benign based on a stable clinical and videodermoscopic follow-up. On dermoscopy, brown structureless areas with regularly arranged globules (white arrows) are visible. (**c**) Spitz nevus of the left helix (inset). Dermoscopy reveals a brown structureless area, peripheral streaks (red arrows) and a centrally located pseudonetwork with ‘red circles’ (white arrows). (**d**) Compound nevus of the right lobule (inset). Dermoscopy shows a pseudonetwork with a centrally located small area of regression (red arrow). Adjacent to the regression area, irregular brown globules are observed (white arrows). (**e**) Blue nevus of the left helix (inset-black arrow). Dermoscopically, the lesion manifests with a blue-greyish structureless area with ill-defined borders. (**f**) Blue nevus of the right scaphoid fossa (inset). A central, structureless, white-grey-brownish area with small grey circles (black arrows) and peripheral grey-brownish hue forming a fine pseudonetwork (red arrows) is seen on dermoscopy. (**a**,**b**,**c**,**e**) FotoFinder, Medicam 800HD [FotoFinder Systems GmbH, Bad Birnbach, Germany]; 20× magnification, immersion gel. (**d**) DermLiteCam [3Gen, San Juan Capistrano, CA, USA]; 10× magnification, polarized light. (**f**) DermLite DL4 [3Gen, San Juan Capistrano, CA, USA] attached to an iPhone 11 [Apple, Cupertino, CA, USA]; 10× magnification, polarized light.

The interobserver agreement for dermoscopic features evaluation was high (Cohen’s kappa score ranging between 0.68–0.81). Clinical and dermoscopic images of selected benign and malignant EEMLs are shown in Figure 3 and Figure 4.

The accuracy of the selected dermoscopic screening algorithms was moderate; sensitivity ranged between 10.7% and 92.9%, and specificity ranged between 58.4% and 93.3%. The summary of the algorithms’ performance is presented in Table 3.

### 3.3. Performance of the Predictive Model

**Figure 5:**
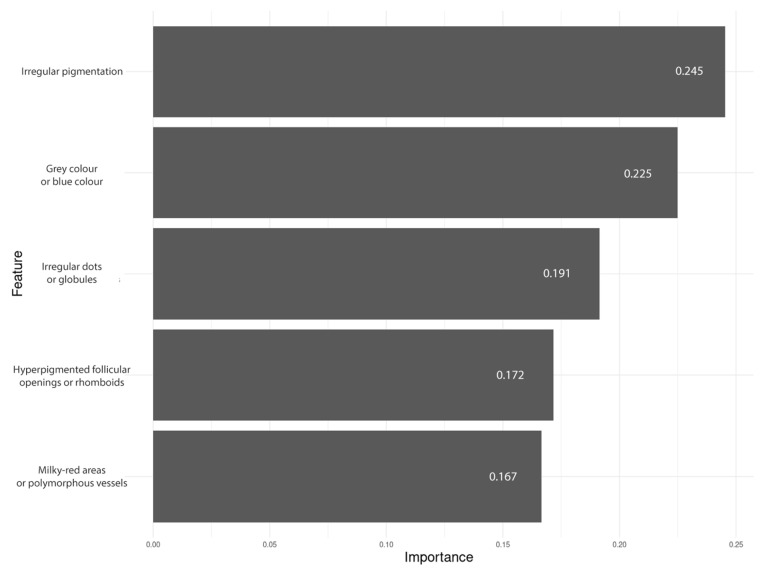
Feature importance plot of the classifier.

The dermoscopic features ultimately included in the predictive model were irregular pigmentation, the presence of grey or blue colour, irregular dots or globules, hyperpigmented follicular openings or rhomboid structures, and milky-red areas or polymorphous vessels. Feature importance ranged from 16.7% to 24.5% (Figure 5).

The final XGBoost classifier achieved a balanced accuracy of 89.5% on the validation set, with a sensitivity of 94.4% and a specificity of 84.6%. The positive predictive value (PPV) was 81%, and the negative predictive value (NPV) was 95.7%. A summary of the model’s performance along with its comparison against other algorithms is presented in Table 3.

ROC curves illustrating the model’s performance on the training, validation, and test sets are provided in Supplementary Figure S1. The file with the predictive model is provided in Supplementary Text S2, and the AuriCheck calculator is available online at https://auricheck.gumed.edu.pl.

## 4. Discussion

This is the first large-scale study providing the clinical and dermoscopic features of EEMLs.

Consistent with existing epidemiological data [25,26], our findings indicate that EEM predominantly affects males, with a male-to-female ratio of 1.67:1, although this gender difference is not statistically significant. The protective role of hair in females, who generally have greater hair coverage than males, is proposed to be accountable for this EEM gender discrepancy [27]. The anatomic distribution of EEMLs also reflects ultraviolet radiation reaching the skin. Both in our dataset and in other studies [8,12,16], the majority of EEMLs were located on the helix, which is the most UV-exposed part of the external ear. On the other hand, the lobule was the only sub-anatomical location where malignant lesions occurred significantly more frequently than benign lesions. Increased age was associated with greater risk of malignancy, which is consistent with other studies [16]. Moreover, the mean diameter of malignant lesions was markedly greater compared to benign lesions.

Lentigo maligna melanoma (LMM) represented the predominant histopathological subtype of EEM, substantially surpassing the second most frequent diagnosis, which was superficial spreading melanoma (SSM). This finding differs from the existing literature, which generally outlines an inverse order [25,28]. Our study incorporated clinical and dermoscopic data from four different regions, which contributed to the constitution of a diverse and, hence, more representative study group. Most of the analysed malignant cases were diagnosed at an early phase (Breslow thickness ≤1.0 mm), enabling the extrapolation of the collected dermoscopic data for early EEM detection.

Comparative analysis of benign and malignant lesions revealed significant differences in the majority of investigated dermoscopic features. A multicolour nature was evident in malignant lesions, with significant variations in black, white, red, blue and grey colours seen under dermoscopy. Traditionally, a grey colour is considered one of the clues for malignancy for early facial melanoma, and indeed, it was one of the predictors of lesion excision. However, in a later subanalysis of melanoma vs. excised nevi it was not significantly associated with melanoma diagnosis. Furthermore, a comparative analysis of the prevalence of a grey colour is not feasible as this feature was not evaluated in most other studies on EEMLs with the exception of Deinlein et al. [16], whose analysis was restricted to the ‘grey-blue colour’ only.

Polymorphic vessels are another general dermoscopic feature suggestive of melanoma, and their significance in differentiating EEMLs was established in our study. The presence of two or more types of vessels, rather than a single type, was an important clue for malignancy.

Interestingly, in our study, a pigmented network was observed much less frequently than a pseudonetwork (pseudo-reticulation). The low prevalence of pigmented network among EEMLs may result from histopathological peculiarities of the skin covering the external ear, manifesting, inter alia, by a relatively thin epidermis and flat rete ridges.

The literature is inconclusive regarding the predominant dermoscopic structures prevailing in EEMs. Consistent with our findings, Kamińska-Winciorek et al. [12] indicated the coexistence of both facial and extra-facial dermoscopic traits in EEMs, whereas Deinlein et al. [16] concluded that face-specific criteria for melanoma should be applied in dermoscopic evaluation of EEMLs. Our results only partially correspond to those obtained by Deinlein et al. [16], who in a smaller study on EEMLs (25 EEMs vs. 18 nevi) also demonstrated asymmetric pigmented follicles and an annular-granular pattern to be associated with EEM. Remarkably, one of the most impactful dermoscopic feature of EEMs in our material—milky red areas—was far from achieving statistical significance in their cohort. In addition, we observed more differences, e.g., lower prevalence of tan peripheral structureless areas and scar-like depigmentation in EEM, and the less frequent presence of regular globules among benign nevi in our material. Observed discrepancies may stem from the distinct methodologies employed in both studies and the difference in mean Breslow thickness between the samples of the respective studies.

As previously undescribed structure and pattern have been observed in the current study, namely ‘red circles’ and ‘annular-globular pattern’, we therefore re-screened figures in the previous papers on EEML for their presence. ‘Red circles’ were visible in one of the figures of the article published by Pajak et al. [29] and Rishpon et al. [8], albeit they were not mentioned by the authors. The ‘annular-globular pattern’ was found in one case of external ear nevus reported by Rishpon et al. [8], yet again this pattern was not described in their study.

We assume that the ‘red circles’ histopathologically correspond to blood vessels, which are concentrically arranged around the hair follicles ostia as presented in the dermoscopic-histopathologic correlation depicted in Figure 1. Based on the histopathological images of lesions presenting the ‘annular-globular’ pattern, we extrapolate that the globules observed in dermoscopy represent large diameter melanocytic nests located around hair follicles, as shown in Figure 1.

Perifollicular linear projections (PLP) are defined as “pigmented short or long lines emanating from and connecting to hair follicles” that, according to Navarrete-Dechent et al. [17], correspond with epidermal projections, composed of pigmented keratinocytes and lentiginous proliferation of melanocytes, emanating outward from dilated follicular infundibula. In a recently published paper, PLP have been demonstrated to be highly specific for head and neck melanoma (specificity = 96%, sensitivity = 61.8%); however, they were much less frequently encountered in other entities (less than 4% of cases). The significance of PLP in EEMLs has not been previously evaluated on a large dataset. Navarrete-Dechent et al. [17] assessed 11 pigmented macules on the ear for PLP, detecting them in three lesions (3/11; 27.3%). We hypothesize that a circle with lines may represent a variant of PLP observed in EEMLs. However, further investigation is warranted, as within our dataset PLP was more frequently identified in benign lesions (4/89; 4.5%) than in melanomas (one LMM case, Breslow thickness = 0.2 mm).

The sensitivity of the analysed algorithms on the training set ranged between 10.5% and 97.4%, and specificity hovered between 57.1% and 95.2%. The anticipated high sensitivity of the only dermoscopic algorithm dedicated to the assessment of facial pigmented lesions—the ‘7-point checklist to rule out facial melanoma’—proved to be the lowest among the models analysed, with a sensitivity slightly exceeding 10%. This may be due to the high prevalence of brown structureless areas in our dataset. The relatively low sensitivity of the 3-point checklist algorithm may be explained by the infrequent presence of the pigmented network. Since none of the analysed algorithms attained adequate sensitivity and specificity, we decided to develop a new predictive model.

The proposed algorithm outperformed existing models, though not all differences were statistically significant. Our model achieved 84.2% sensitivity and 90.5% specificity on a training set, and reached 90% sensitivity and 86.7% specificity on an independent test set. On the test set, the other algorithms either maintained high sensitivity with low specificity (7-point checklist, ‘Chaos and Clues’, Menzies method) or reconciled moderate sensitivity with high specificity (3-point checklist, 7-non melanoma features to rule out melanoma, CASH algorithm). The seemingly high performance of the analysed algorithms on the test set and subsequent modest statistical differences with our model may have resulted from the limited number of cases in the test set. As no dermoscopic algorithm for EEMLs had been developed so far, we proposed a predictive model that further augments the parameters of the already existing algorithms yet is approachable enough for rapid assessment of EEMLs in routine clinical practice.

This study has intrinsic limitations due to the retrospective character of the study design. Benign melanocytic lesions that were both stable during the follow-up period and exhibited unequivocal dermoscopy represent the spectrum of frequently encountered cases in everyday clinical practice, and were hence included in the final analysis to avoid selection bias. As not all of the videodermoscopic images were captured in the polarized mode, features visible in the polarized mode only (e.g., rosettes) were not included in the analysis, which could negatively contribute to the overall study bias. Moreover, the lack of complete demographic data and information on histopathological subtypes precluded the comprehensive correlation and prospective integration of certain variables into the algorithm. Another limitation was the relatively low number of both benign and malignant auricular lesions found in the HAM10000 database (15 and 10 cases, respectively). Such a scant test set significantly impaired the validation of our predictive model, necessitating its further evaluation on a separate dataset. Dermoscopy findings of skin tumours exhibit certain variations depending on the phototype [30]. Since our study included patients with Fitzpatrick skin phototypes I–III, the results cannot be generalized to the entire population. Another source of potential bias was the disproportionately high number of melanomas in the analysed cohort compared to their actual prevalence in the clinical setting. This disproportion is a consequence of excluding clearly benign lesions from the analysis and considering only those that raised oncological suspicion, necessitating either surgical removal or prolonged videodermoscopic follow-up.

## 5. Conclusions

Dermoscopy of EEM shares the features characteristic of both “facial” and “non-facial” melanoma. As known dermoscopic screening algorithms perform moderately well when it comes to the assessment of EEMLs, the proposed model may serve as an alternative for EEM detection. Additionally, we described an additional dermoscopic feature — the ‘red circles’ that seem to be specific to external ear malignancy — and a previously unreported dermoscopic pattern — the ‘annular-globular pattern’. However, as this is the first comprehensive study on the dermoscopic features of EEMLs, the performance of our model and the occurrence of the ‘red circles’ and the ‘annular-globular pattern’ in both the external ear and other anatomical regions requires further validation and assessment on other datasets.

## Figures and Tables

**Table 1:** Study group’s demographics and lesions’ clinical characteristics.

	Malignant			Benign					Total Malignant vs. Total Benign *p* Value	Total Malignant vs. Excised Benign Lesions ^6^ *p* Value	Total Melanoma vs. Nevi Excised *p* Value
	Melanoma ^1^ (*n* = 48)	Other Entities ^2^ (*n* = 8)	Total Malignant (*n* = 56)	Nevi Excised ^3^ (*n* = 37)	Benign Melanocytic Nevi— Followed-Up ^4^ (*n* = 40)	Spitz Nevus (*n* = 6)	Other Entities ^5^ (*n* = 6)	Total Benign (*n* = 89)			
**Female, No (%)**	18/48; 37.5%	4/8; 50%	22/56; 39.3%	19/37; 51.4%	21/40; 52.5%	0/6; 0%	5/6; 83.3%	45/89; 50.6%	0.2315	0.3324	0.2704
**Age at diagnosis, mean years ± SD (range)**	61.7 ± 18.3 (7–88)	37.5 ± 28.6 (4–80)	58.3 ± 21.6 (4–88)	40.2 ± 17.1 (11–72)	37.9 ± 13.1 (19–74)	21.8 ± 20.4 (7–54)	37.2 ± 20.8 (17–74)	37.7 ± 16.2 (7–74)	<0.0001 ***	<0.0001 ***	<0.0001 ***
**Followed-up** **lesions,** **No (%)**	4/48; 8.3%	2/8; 25%	6/56; 10.7%	6/37; 16.2%	40/40; 100%	1/6; 16.7%	2/6; 33.3%	49/89; 55.1%	<0.0001 ***	0.2806	0.3196
**Follow-up** **period,** **mean months ± SD (range)**	33 ± 35.1 (8–83)	29 ± 32.5 (6–52)	31.7 ± 30.9 (6–83)	18.3 ± 13.3 (4–35)	45 ± 31.9 (24–157)	8 ± NA (8–8)	32.5 ± 38.9 (5–60)	40.4 ± 31.3 (4–157)	0.7232	0.7232	1
**Anatomical location, No (%)**											
**Antihelix**	0/48; 0%	0/8; 0%	0/56; 0%	1/37; 2.7%	0/40; 0%	1/6; 16.7%	1/6; 16.7%	3/89; 3.4%	0.2839	0.0983	0.4353
**Antitragus**	3/48; 6.2%	0/8; 0%	3/56; 5.4%	2/37; 5.4%	5/40; 12.5%	0/6; 0%	0/6; 0%	7/89; 7.9%	0.7411	1	1
**Concha**	0/48; 0%	0/8; 0%	0/56; 0%	1/37; 2.7%	1/40; 2.5%	0/6; 0%	1/6; 16.7%	3/89; 3.4%	0.2839	0.2154	0.4353
**Crus of antihelix**	0/48; 0%	0/8; 0%	0/56; 0%	2/37; 5.4%	1/40; 2.5%	0/6; 0%	0/6; 0%	3/89; 3.4%	0.2839	0.2154	0.1866
**Crus of helix**	1/48; 2.1%	0/8; 0%	1/56; 1.8%	0/37; 0%	0/40; 0%	0/6; 0%	0/6; 0%	0/89; 0%	0.3862	1	1
**Helix**	26/48; 54.2%	5/8; 62.5%	31/56; 55.4%	19/37; 51.4%	23/40; 57.5%	5/6; 83.3%	3/6; 50%	50/89; 56.2%	1	1	0.8294
**Lobule**	11/48; 22.9%	2/8; 25%	13/56; 23.2%	4/37; 10.8%	4/40; 10%	0/6; 0%	0/6; 0%	8/89; 9%	0.0276 *	0.0609	0.1657
**Posterior part of auricle**	0/48; 0%	1/8; 12.5%	1/56; 1.8%	3/37; 8.1%	0/40; 0%	0/6; 0%	0/6; 0%	3/89; 3.4%	1	0.337	0.0787
**Scaphoid fossa**	5/48; 10.4%	0/8; 0%	5/56; 8.9%	5/37; 13.5%	6/40; 15%	0/6; 0%	1/6; 16.7%	12/89; 13.5%	0.5969	0.7515	0.741
**Tragus**	2/48; 4.2%	0/8; 0%	2/56; 3.6%	0/37; 0%	0/40; 0%	0/6; 0%	0/6; 0%	0/89; 0%	0.1475	0.4974	0.5025
**Left side,** **No (%)**	26/48; 54.2%	3/8; 37.5%	29/56; 51.8%	18/37; 48.6%	21/40; 52.5%	4/6; 66.7%	2/6; 33.3%	45/89; 50.6%	1	0.8459	0.6654
**Lesion diameter, mean millimetres ± SD (range)**	11.2 ± 8 (3.2–50)	6.4 ± 2.6 (3.4–11.1)	10.5 ± 7.6 (3.2–50)	5.3 ± 3 (1.6–17)	4.2 ± 1.6 (1.7–7.6)	4 ± 1.2 (2.1–5.2)	5.4 ± 3.2 (1.4–9)	4.7 ± 2.4 (1.4–17)	<0.0001 ***	<0.0001 ***	<0.0001 ***

^1^ In situ melanoma-11/47 (23.4%); melanoma of Breslow thickness ≤1.0 mm–27/47 (57.4%); melanoma of Breslow thickness >1.0 mm–9/47 (19.1%). The mean Breslow thickness of invasive melanomas was 0.76 ± 0.59 mm. Histopathological melanoma subtypes: lentigo maligna melanoma – 25/47 (53.2%); superficial spreading melanoma-16/47 (34%); desmoplastic melanoma–1/47 (2.1%); melanoma in nevo-1/47 (2.1%); nevoid melanoma-1/47 (2.1%); nodular melanoma–1/47 (2.1%); spindle cell melanoma-1/47 (2.1%); Spitzoid melanoma-1/47 (2.1%). Melanoma subtype and Breslow thickness of one melanoma case could not be evaluated. ^2^ MELTUMP (*n* = 3), SAMPUS (*n* = 2), Atypical Spitz tumour (*n* = 2), IAMPUS (*n* = 1). ^3^ Compound nevus (*n* = 15), dermal nevus (*n* = 12), junctional nevus (*n* = 8), dysplastic nevus (*n* = 2). ^4^ At least 24 months of stable clinical and videodermoscopic follow-up. ^5^ Blue nevus (*n* = 3), solar lentigo (*n* = 3).^6^ Nevi excised, Spitz nevi, other entities. N/A-not applicable, SD-standard deviation. * *p* < 0.05, *** *p* < 0.001.

**Table 2:** Dermoscopic findings of malignant and benign melanocytic lesions located on the external ear.

Dermoscopic Features		Malignant			Benign							
		Melanoma (*n* = 48)	Other Entities (*n* = 8)	Total Malignant (*n* = 56)	Nevi Excised (*n* = 37)	Benign Melanocytic Nevi—Followed-Up (*n* = 40)	Spitz Nevus (*n* = 6)	Other Entities (*n* = 6)	Total Benign (*n* = 89)	Total Malignant vs. Total Benign *p* Value	Total Malignant vs. Excised Benign Lesions *p* Value	Total Melanoma vs. Nevi Excised *p* Value
**Presence of colour**	Light brown	35/48; 72.9%	6/8; 75%	41/56; 73.2%	30/37; 81.1%	37/40; 92.5%	3/6; 50%	2/6; 33.3%	72/89; 80.9%	0.3078	1	0.4459
	Dark brown	42/48; 87.5%	7/8; 87.5%	49/56; 87.5%	34/37; 91.9%	23/40; 57.5%	6/6; 100%	3/6; 50%	66/89; 74.2%	0.0604	1	0.7253
	Black	17/48; 35.4%	0/8; 0%	17/56; 30.4%	2/37; 5.4%	0/40; 0%	0/6; 0%	0/6; 0%	2/89; 2.2%	<0.0001 ***	0.0006 ***	0.0012 **
	White	31/48; 64.6%	4/8; 50%	35/56; 62.5%	6/37; 16.2%	0/40; 0%	1/6; 16.7%	1/6; 16.7%	8/89; 9%	<0.0001 ***	<0.0001 ***	<0.0001 ***
	Grey	29/48; 60.4%	5/8; 62.5%	34/56; 60.7%	14/37; 37.8%	3/40; 7.5%	5/6; 83.3%	2/6; 33.3%	24/89; 27%	0.0001 ***	0.08	0.05
	Red	35/48; 72.9%	7/8; 87.5%	42/56; 75%	8/37; 21.6%	1/40; 2.5%	2/6; 33.3%	0/6; 0%	11/89; 12.4%	<0.0001 ***	<0.0001 ***	<0.0001 ***
	Blue	9/48; 18.8%	1/8; 12.5%	10/56; 17.9%	1/37; 2.7%	0/40; 0%	0/6; 0%	3/6; 50%	4/89; 4.5%	0.0177 *	0.1638	0.0379 *
**Total number of colours,** **mean number ± SD (range)**		4.1 ± 1.3 (1–6)	3.8 ± 1.3 (2–5)	4.1 ± 1.3 (1–6)	2.6 ± 1.1 (1–5)	1.6 ± 0.6 (1–3)	2.8 ± 0.4 (2–3)	1.8 ± 1 (1–3)	2.1 ± 1 (1–5)	<0.0001 ***	<0.0001 ***	<0.0001 ***
**Features** **characteristic for facial** **melanoma**	Annular granular pattern	11/48; 22.9%	1/8; 12.5%	12/56; 21.4%	1/37; 2.7%	0/40; 0%	0/6; 0%	0/6; 0%	1/89; 1.1%	<0.0001 ***	0.0025 **	0.0102 *
	Asymmetric pigmented follicular openings	30/48; 62.5%	4/8; 50%	34/56; 60.7%	15/37; 40.5%	2/40; 5%	1/6; 16.7%	0/6; 0%	18/89; 20.2%	<0.0001 ***	0.0059 **	0.0516
	Circle within a circle	8/48; 16.7%	0/8; 0%	8/56; 14.3%	0/37; 0%	0/40; 0%	0/6; 0%	0/6; 0%	0/89; 0%	0.0004 ***	0.0067 **	0.0086 **
	Dark blotches and obliterated hair follicles	18/48; 37.5%	1/8; 12.5%	19/56; 33.9%	5/37; 13.5%	1/40; 2.5%	3/6; 50%	0/6; 0%	9/89; 10.1%	0.0009 ***	0.0462 *	0.0152 *
	Hyperpigmented follicular openings	32/48; 66.7%	4/8; 50%	36/56; 64.3%	16/37; 43.2%	2/40; 5%	2/6; 33.3%	0/6; 0%	20/89; 22.5%	<0.0001 ***	0.0062 **	0.0466 *
	Increased density of the vascular network	34/48; 70.8%	3/8; 37.5%	37/56; 66.1%	11/37; 29.7%	3/40; 7.5%	0/6; 0%	0/6; 0%	14/89; 15.7%	<0.0001 ***	<0.0001 ***	0.0002 ***
	Polygons/zig zag	24/48; 50%	3/8; 37.5%	27/56; 48.2%	10/37; 27%	0/40; 0%	1/6; 16.7%	0/6; 0%	11/89; 12.4%	<0.0001 ***	0.0081 **	0.0445 *
	Red rhomboidal structures	12/48; 25%	0/8; 0%	12/56; 21.4%	0/37; 0%	0/40; 0%	1/6; 16.7%	0/6; 0%	1/89; 1.1%	<0.0001 ***	0.0025 **	0.0009 ***
	Rhomboids	23/48; 47.9%	3/8; 37.5%	26/56; 46.4%	7/37; 18.9%	2/40; 5%	1/6; 16.7%	0/6; 0%	10/89; 11.2%	<0.0001 ***	0.0015 **	0.0064 **
**Total number of features characteristic for facial melanoma, mean number ± SD (range)**		4.3 ± 2.6 (0–8)	2.5 ± 2.1 (0–5)	4.1 ± 2.6 (0–8)	2.1 ± 2 (0–6)	0.7 ± 0.9 (0–4)	1.8 ± 2.1 (0–6)	0.5 ± 0.5 (0–1)	1.3 ± 1.6 (0–6)	<0.0001 ***	0.0001 ***	<0.0001 ***
**Features** **characteristic for non-facial melanoma**	Irregular globules	22/48; 45.8%	4/8; 50%	26/56; 46.4%	15/37; 40.5%	3/40; 7.5%	2/6; 33.3%	0/6; 0%	20/89; 22.5%	0.0034 **	0.2396	0.6643
	Irregular pigmentation	44/48; 91.7%	6/8; 75%	50/56; 89.3%	18/37; 48.6%	2/40; 5%	1/6; 16.7%	0/6; 0%	21/89; 23.6%	<0.0001 ***	<0.0001 ***	<0.0001 ***
	Milky-red areas	32/48; 66.7%	7/8; 87.5%	39/56; 69.6%	4/37; 10.8%	0/40; 0%	1/6; 16.7%	0/6; 0%	5/89; 5.6%	<0.0001 ***	<0.0001 ***	<0.0001 ***
	Multiple small hyperpigmented areas/patchy pigmented islands	11/48; 22.9%	3/8; 37.5%	14/56; 25%	9/37; 24.3%	4/40; 10%	1/6; 16.7%	0/6; 0%	14/89; 15.7%	0.1974	0.6455	1
	Negative network	6/48; 12.5%	0/8; 0%	6/56; 10.7%	0/37; 0%	0/40; 0%	0/6; 0%	0/6; 0%	0/89; 0%	0.0028 **	0.0289 *	0.0334 *
	Regression erythema/vessels/scar-like depigmentation	12/48; 25%	2/8; 25%	14/56; 25%	1/37; 2.7%	0/40; 0%	0/6; 0%	0/6; 0%	1/89; 1.1%	<0.0001 ***	0.0006 ***	0.0052 **
	Regression granularity/peppering	5/48; 10.4%	1/8; 12.5%	6/56; 10.7%	0/37; 0%	0/40; 0%	0/6; 0%	0/6; 0%	0/89; 0%	0.0028 **	0.0289 *	0.0655
	Regular globules	2/48; 4.2%	0/8; 0%	2/56; 3.6%	5/37; 13.5%	7/40; 17.5%	0/6; 0%	0/6; 0%	12/89; 13.5%	0.0801	0.2473	0.2312
	Tan peripheral structureless area	2/48; 4.2%	0/8; 0%	2/56; 3.6%	1/37; 2.7%	3/40; 7.5%	0/6; 0%	0/6; 0%	4/89; 4.5%	1	1	1
	Atypical network	3/48; 6.2%	0/8; 0%	3/56; 5.4%	2/37; 5.4%	1/40; 2.5%	0/6; 0%	0/6; 0%	3/89; 3.4%	0.6765	1	1
	Blue-white veil	7/48; 14.6%	0/8; 0%	7/56; 12.5%	1/37; 2.7%	0/40; 0%	0/6; 0%	0/6; 0%	1/89; 1.1%	0.0055 **	0.0647	0.13
	Irregular streaks	2/48; 4.2%	1/8; 12.5%	3/56; 5.4%	0/37; 0%	0/40; 0%	1/6; 16.7%	0/6; 0%	1/89; 1.1%	0.2986	0.6211	0.5025
	Irregular blotches [Black, brown and ⁄ or grey structureless areas asymmetrically distributed within the lesion]	11/48; 22.9%	2/8; 25%	13/56; 23.2%	8/37; 21.6%	1/40; 2.5%	0/6; 0%	0/6; 0%	9/89; 10.1%	0.0551	0.4663	1
**Total number of features** **characteristic for non-facial melanoma,** **mean number ± SD (range)**		3.3 ± 1.5 (0–7)	3.2 ± 1.4 (1–5)	3.3 ± 1.5 (0–7)	1.7 ± 1.4 (0–4)	0.5 ± 1 (0–5)	1 ± 0.9 (0–2)	0 ± 0 (0–0)	1 ± 1.3 (0–5)	<0.0001 ***	<0.0001 ***	<0.0001 ***
**Vascular structures**	Presence of vessels	33/48; 68.8%	7/8; 87.5%	40/56; 71.4%	12/37; 32.4%	4/40; 10%	1/6; 16.7%	0/6; 0%	17/89; 19.1%	<0.0001 ***	<0.0001 ***	0.0011 **
	Milky-red areas/globules	32/48; 66.7%	7/8; 87.5%	39/56; 69.6%	4/37; 10.8%	0/40; 0%	1/6; 16.7%	0/6; 0%	5/89; 5.6%	<0.0001 ***	<0.0001 ***	<0.0001 ***
	Coiled (glomerular) vessels	2/48; 4.2%	0/8; 0%	2/56; 3.6%	1/37; 2.7%	0/40; 0%	0/6; 0%	0/6; 0%	1/89; 1.1%	0.5593	1	1
	Looped (hairpin) vessels	0/48; 0%	0/8; 0%	0/56; 0%	0/37; 0%	0/40; 0%	0/6; 0%	0/6; 0%	0/89; 0%	-	-	-
	Linear irregular (serpentine) vessels	23/48; 47.9%	2/8; 25%	25/56; 44.6%	7/37; 18.9%	0/40; 0%	0/6; 0%	0/6; 0%	7/89; 7.9%	<0.0001 ***	0.0012 **	0.0064 **
	Helical (corkscrew) vessels	0/48; 0%	0/8; 0%	0/56; 0%	0/37; 0%	0/40; 0%	0/6; 0%	0/6; 0%	0/89; 0%	-	-	-
	Curved (comma) vessels	0/48; 0%	0/8; 0%	0/56; 0%	2/37; 5.4%	0/40; 0%	0/6; 0%	0/6; 0%	2/89; 2.2%	0.5226	0.2154	0.1866
	Dotted vessels	7/48; 14.6%	2/8; 25%	9/56; 16.1%	5/37; 13.5%	4/40; 10%	0/6; 0%	0/6; 0%	9/89; 10.1%	0.3105	0.4068	1
	Monomorphous vessels (exactly 1 type of vessel)	9/48; 18.8%	4/8; 50%	13/56; 23.2%	8/37; 21.6%	4/40; 10%	1/6; 16.7%	0/6; 0%	13/89; 14.6%	0.2659	0.6339	0.7891
	Polymporphous vessels (>1 type of vessel)	24/48; 50%	3/8; 37.5%	27/56; 48.2%	4/37; 10.8%	0/40; 0%	0/6; 0%	0/6; 0%	4/89; 4.5%	<0.0001 ***	<0.0001 ***	0.0002 ***
**SELECTED DERMOSCOPIC ALGORITHMS**												
**7-non** **melanoma** **features to rule out melanoma**	Scales (pigmented or non-pigmented)	4/48; 8.3%	1/8; 12.5%	5/56; 8.9%	1/37; 2.7%	0/40; 0%	0/6; 0%	0/6; 0%	1/89; 1.1%	0.032 *	0.2115	0.3816
	White follicles (white circles, follicular white clods and/or 4 dots clods (rosettes))	11/48; 22.9%	0/8; 0%	11/56; 19.6%	0/37; 0%	0/40; 0%	0/6; 0%	0/6; 0%	0/89; 0%	<0.0001 ***	0.0007 ***	0.0019 **
	Erythema/reticular vessels	32/48; 66.7%	5/8; 62.5%	37/56; 66.1%	5/37; 13.5%	0/40; 0%	2/6; 33.3%	0/6; 0%	7/89; 7.9%	<0.0001 ***	<0.0001 ***	<0.0001 ***
	Reticular lines/parallel lines (fingerprints)	3/48; 6.2%	0/8; 0%	3/56; 5.4%	1/37; 2.7%	5/40; 12.5%	0/6; 0%	0/6; 0%	6/89; 6.7%	1	0.6211	0.629
	Brown structureless	27/48; 56.2%	5/8; 62.5%	32/56; 57.1%	27/37; 73%	33/40; 82.5%	5/6; 83.3%	4/6; 66.7%	69/89; 77.5%	0.0153 *	0.1024	0.1723
	Sharp demarcation	2/48; 4.2%	1/8; 12.5%	3/56; 5.4%	7/37; 18.9%	2/40; 5%	2/6; 33.3%	0/6; 0%	11/89; 12.4%	0.2487	0.062	0.0371 *
	Seborrheic keratosis features	0/48; 0%	0/8; 0%	0/56; 0%	1/37; 2.7%	0/40; 0%	0/6; 0%	0/6; 0%	1/89; 1.1%	1	0.4667	0.4353
**Positive test**		6/48; 12.5%	0/8; 0%	6/56; 10.7%	6/37; 16.2%	6/40; 15%	0/6; 0%	2/6; 33.3%	14/89; 15.7%	0.465	0.5664	0.7561
**7-point** **checklist**	Atypical network	3/48; 6.2%	0/8; 0%	3/56; 5.4%	2/37; 5.4%	1/40; 2.5%	0/6; 0%	0/6; 0%	3/89; 3.4%	0.6765	1	1
	Blue-white veil	7/48; 14.6%	0/8; 0%	7/56; 12.5%	1/37; 2.7%	0/40; 0%	0/6; 0%	0/6; 0%	1/89; 1.1%	0.0055 **	0.0647	0.13
	Atypical vascular pattern [Linear-irregular vessels, dotted vessels and ⁄ or milky-red areas not clearly seen within regression structures]	33/48; 68.8%	7/8; 87.5%	40/56; 71.4%	10/37; 27%	4/40; 10%	1/6; 16.7%	0/6; 0%	15/89; 16.9%	<0.0001 ***	<0.0001 ***	0.0002 ***
	Irregular dots ⁄globules	31/48; 64.6%	4/8; 50%	35/56; 62.5%	18/37; 48.6%	6/40; 15%	2/6; 33.3%	0/6; 0%	26/89; 29.2%	0.0001 ***	0.0322 *	0.1849
	Irregular streaks	2/48; 4.2%	1/8; 12.5%	3/56; 5.4%	0/37; 0%	0/40; 0%	1/6; 16.7%	0/6; 0%	1/89; 1.1%	0.2986	0.6211	0.5025
	Irregular blotches [Black, brown and ⁄ or grey structureless areas asymmetrically distributed within the lesion]	11/48; 22.9%	2/8; 25%	13/56; 23.2%	8/37; 21.6%	1/40; 2.5%	0/6; 0%	0/6; 0%	9/89; 10.1%	0.0551	0.4663	1
	Regression structures	13/48; 27.1%	2/8; 25%	15/56; 26.8%	1/37; 2.7%	0/40; 0%	0/6; 0%	0/6; 0%	1/89; 1.1%	<0.0001 ***	0.0003 ***	0.0026 **
**Positive test**		44/48; 91.7%	8/8; 100%	52/56; 92.9%	23/37; 62.2%	10/40; 25%	4/6; 66.7%	0/6; 0%	37/89; 41.6%	<0.0001 ***	<0.0001 ***	0.0013 **
**Chaos and clues**	Asymmetry of pattern	42/48; 87.5%	7/8; 87.5%	49/56; 87.5%	17/37; 45.9%	5/40; 12.5%	1/6; 16.7%	0/6; 0%	23/89; 25.8%	<0.0001 ***	<0.0001 ***	<0.0001 ***
	Asymmetry of colour	43/48; 89.6%	5/8; 62.5%	48/56; 85.7%	18/37; 48.6%	5/40; 12.5%	1/6; 16.7%	0/6; 0%	24/89; 27%	<0.0001 ***	<0.0001 ***	<0.0001 ***
	Grey or blue structures	33/48; 68.8%	5/8; 62.5%	38/56; 67.9%	14/37; 37.8%	3/40; 7.5%	5/6; 83.3%	3/6; 50%	25/89; 28.1%	<0.0001 ***	0.0292 *	0.0079 **
	Thick lines reticular or branched	3/48; 6.2%	0/8; 0%	3/56; 5.4%	1/37; 2.7%	5/40; 12.5%	0/6; 0%	0/6; 0%	6/89; 6.7%	1	0.6211	0.629
	White lines	15/48; 31.2%	2/8; 25%	17/56; 30.4%	1/37; 2.7%	0/40; 0%	0/6; 0%	0/6; 0%	1/89; 1.1%	<0.0001 ***	<0.0001 ***	0.0006 ***
	Eccentric structureless areas	12/48; 25%	1/8; 12.5%	13/56; 23.2%	3/37; 8.1%	2/40; 5%	0/6; 0%	0/6; 0%	5/89; 5.6%	0.0034 **	0.0271 *	0.0496 *
	Peripheral black dots or clods	3/48; 6.2%	0/8; 0%	3/56; 5.4%	0/37; 0%	0/40; 0%	0/6; 0%	0/6; 0%	0/89; 0%	0.0557	0.2462	0.2538
	Radial lines or pseudopods, segmental	3/48; 6.2%	1/8; 12.5%	4/56; 7.1%	2/37; 5.4%	0/40; 0%	5/6; 83.3%	0/6; 0%	7/89; 7.9%	1	0.3399	1
	Polymorphous vessels	24/48; 50%	3/8; 37.5%	27/56; 48.2%	4/37; 10.8%	0/40; 0%	0/6; 0%	0/6; 0%	4/89; 4.5%	<0.0001 ***	<0.0001 ***	0.0002 ***
	Polygons/zig zag	24/48; 50%	3/8; 37.5%	27/56; 48.2%	10/37; 27%	0/40; 0%	1/6; 16.7%	0/6; 0%	11/89; 12.4%	<0.0001 ***	0.0081 **	0.0445 *
**Positive test**		44/48; 91.7%	6/8; 75%	50/56; 89.3%	17/37; 45.9%	5/40; 12.5%	0/6; 0%	0/6; 0%	22/89; 24.7%	<0.0001 ***	<0.0001 ***	<0.0001 ***
**CASH** **algorithm**	Light brown	35/48; 72.9%	6/8; 75%	41/56; 73.2%	30/37; 81.1%	37/40; 92.5%	3/6; 50%	2/6; 33.3%	72/89; 80.9%	0.3078	1	0.4459
	Dark brown	42/48; 87.5%	7/8; 87.5%	49/56; 87.5%	34/37; 91.9%	23/40; 57.5%	6/6; 100%	3/6; 50%	66/89; 74.2%	0.0604	1	0.7253
	Black	17/48; 35.4%	0/8; 0%	17/56; 30.4%	2/37; 5.4%	0/40; 0%	0/6; 0%	0/6; 0%	2/89; 2.2%	<0.0001 ***	0.0006 ***	0.0012 **
	Red	35/48; 72.9%	7/8; 87.5%	42/56; 75%	8/37; 21.6%	1/40; 2.5%	2/6; 33.3%	0/6; 0%	11/89; 12.4%	<0.0001 ***	<0.0001 ***	<0.0001 ***
	White	31/48; 64.6%	4/8; 50%	35/56; 62.5%	6/37; 16.2%	0/40; 0%	1/6; 16.7%	1/6; 16.7%	8/89; 9%	<0.0001 ***	<0.0001 ***	<0.0001 ***
	Blue	9/48; 18.8%	1/8; 12.5%	10/56; 17.9%	1/37; 2.7%	0/40; 0%	0/6; 0%	3/6; 50%	4/89; 4.5%	0.0177 *	0.1638	0.0379 *
	Architectural disorder [0-none/mild; 1-moderate; 2-marked]	0: 3/48; 6.2% 1: 15/48; 31.2% 2: 30/48; 62.5%	0: 2/8; 25% 1: 3/8; 37.5% 2: 3/8; 37.5%	0: 5/56; 8.9% 1: 18/56; 32.1% 2: 33/56; 58.9%	0: 16/37; 43.2% 1: 16/37; 43.2% 2: 5/37; 13.5%	0: 35/40; 87.5% 1: 5/40; 12.5% 2: 0/40; 0%	0: 4/6; 66.7% 1: 2/6; 33.3% 2: 0/6; 0%	0: 6/6; 100% 1: 0/6; 0% 2: 0/6; 0%	0: 61/89; 68.5% 1: 23/89; 25.8% 2: 5/89; 5.6%	<0.0001 ***	<0.0001 ***	<0.0001 ***
	Symmetry, shape and dermoscopic structures [0-biaxial symmetry; 1-monoaxial symmetry; 2-biaxial asymmetry]	0: 3/48; 6.2% 1: 17/48; 35.4% 2: 28/48; 58.3%	0: 2/8; 25% 1: 3/8; 37.5% 2: 3/8; 37.5%	0: 5/56; 8.9% 1: 20/56; 35.7% 2: 31/56; 55.4%	0: 16/37; 43.2% 1: 17/37; 45.9% 2: 4/37; 10.8%	0: 33/40; 82.5% 1: 7/40; 17.5% 2: 0/40; 0%	0: 4/6; 66.7% 1: 2/6; 33.3% 2: 0/6; 0%	0: 6/6; 100% 1: 0/6; 0% 2: 0/6; 0%	0: 59/89; 66.3% 1: 26/89; 29.2% 2: 4/89; 4.5%	<0.0001 ***	<0.0001 ***	<0.0001 ***
	Network	9/48; 18.8%	0/8; 0%	9/56; 16.1%	5/37; 13.5%	10/40; 25%	0/6; 0%	1/6; 16.7%	16/89; 18%	0.8248	0.7808	0.5696
	Dots/globules	34/48; 70.8%	4/8; 50%	38/56; 67.9%	24/37; 64.9%	17/40; 42.5%	4/6; 66.7%	0/6; 0%	45/89; 50.6%	0.0575	0.313	0.6408
	Streaks/pseudopods	3/48; 6.2%	1/8; 12.5%	4/56; 7.1%	2/37; 5.4%	0/40; 0%	5/6; 83.3%	0/6; 0%	7/89; 7.9%	1	0.3399	1
	Blue-white veil	7/48; 14.6%	0/8; 0%	7/56; 12.5%	1/37; 2.7%	0/40; 0%	0/6; 0%	0/6; 0%	1/89; 1.1%	0.0055 **	0.0647	0.13
	Regression structures [grey areas with or without peppering; scarring]	14/48; 29.2%	2/8; 25%	16/56; 28.6%	1/37; 2.7%	0/40; 0%	0/6; 0%	0/6; 0%	1/89; 1.1%	<0.0001 ***	0.0001 ***	0.0013 **
	Blotches [structureless regions of any colour occupying [10% of the area of the lesion]	12/48; 25%	2/8; 25%	14/56; 25%	9/37; 24.3%	1/40; 2.5%	0/6; 0%	0/6; 0%	10/89; 11.2%	0.039 *	0.4825	1
	Polymorphous blood vessels [including dotted and irregular linear]	24/48; 50%	3/8; 37.5%	27/56; 48.2%	4/37; 10.8%	0/40; 0%	0/6; 0%	0/6; 0%	4/89; 4.5%	<0.0001 ***	<0.0001 ***	0.0002 ***
**Positive test**		34/48; 70.8%	4/8; 50%	38/56; 67.9%	6/37; 16.2%	0/40; 0%	0/6; 0%	0/6; 0%	6/89; 6.7%	<0.0001 ***	<0.0001 ***	<0.0001 ***
**Menzies** **algorithm**	Point and axial symmetry of pigmentation	5/48; 10.4%	3/8; 37.5%	8/56; 14.3%	19/37; 51.4%	33/40; 82.5%	5/6; 83.3%	6/6; 100%	63/89; 70.8%	<0.0001 ***	<0.0001 ***	<0.0001 ***
	Presence of only a single colour	1/48; 2.1%	0/8; 0%	1/56; 1.8%	5/37; 13.5%	18/40; 45%	0/6; 0%	3/6; 50%	26/89; 29.2%	<0.0001 ***	0.0116 *	0.0812
	Blue-white veil	7/48; 14.6%	0/8; 0%	7/56; 12.5%	1/37; 2.7%	0/40; 0%	0/6; 0%	0/6; 0%	1/89; 1.1%	0.0055 **	0.0647	0.13
	Multiple brown dots	21/48; 43.8%	2/8; 25%	23/56; 41.1%	12/37; 32.4%	12/40; 30%	1/6; 16.7%	0/6; 0%	25/89; 28.1%	0.1467	0.1501	0.3705
	Pseudopods	1/48; 2.1%	1/8; 12.5%	2/56; 3.6%	2/37; 5.4%	0/40; 0%	5/6; 83.3%	0/6; 0%	7/89; 7.9%	0.4827	0.0788	0.5774
	Radial streaming [asymmetric]	2/48; 4.2%	1/8; 12.5%	3/56; 5.4%	0/37; 0%	0/40; 0%	1/6; 16.7%	0/6; 0%	1/89; 1.1%	0.2986	0.6211	0.5025
	Scar-like depigmentation	11/48; 22.9%	0/8; 0%	11/56; 19.6%	1/37; 2.7%	0/40; 0%	0/6; 0%	0/6; 0%	1/89; 1.1%	0.0001 ***	0.0049 **	0.0102 *
	Peripheral black dots/globules	3/48; 6.2%	0/8; 0%	3/56; 5.4%	0/37; 0%	0/40; 0%	0/6; 0%	0/6; 0%	0/89; 0%	0.0557	0.2462	0.2538
	Multiple (5–6) colours	22/48; 45.8%	3/8; 37.5%	25/56; 44.6%	2/37; 5.4%	0/40; 0%	0/6; 0%	0/6; 0%	2/89; 2.2%	<0.0001 ***	<0.0001 ***	<0.0001 ***
	Multiple blue/grey dots	11/48; 22.9%	1/8; 12.5%	12/56; 21.4%	0/37; 0%	0/40; 0%	0/6; 0%	0/6; 0%	0/89; 0%	<0.0001 ***	0.0003 ***	0.0019 **
	Broadened network	3/48; 6.2%	0/8; 0%	3/56; 5.4%	2/37; 5.4%	1/40; 2.5%	0/6; 0%	1/6; 16.7%	4/89; 4.5%	1	1	1
**Positive test**		34/48; 70.8%	4/8; 50%	38/56; 67.9%	11/37; 29.7%	4/40; 10%	1/6; 16.7%	0/6; 0%	16/89; 18%	<0.0001 ***	<0.0001 ***	0.0002 ***
**3-point** **checklist**	Asymmetry [Asymmetrical distribution of colours and dermoscopic structures]	43/48; 89.6%	5/8; 62.5%	48/56; 85.7%	16/37; 43.2%	7/40; 17.5%	1/6; 16.7%	0/6; 0%	24/89; 27%	<0.0001 ***	<0.0001 ***	<0.0001 ***
	Atypical network [Pigmented network with irregular holes and thick lines]	3/48; 6.2%	0/8; 0%	3/56; 5.4%	2/37; 5.4%	1/40; 2.5%	0/6; 0%	1/6; 16.7%	4/89; 4.5%	1	1	1
	Blue-white structures [Any type of blue and/or white colour]	32/48; 66.7%	5/8; 62.5%	37/56; 66.1%	6/37; 16.2%	0/40; 0%	1/6; 16.7%	3/6; 50%	10/89; 11.2%	<0.0001 ***	<0.0001 ***	<0.0001 ***
**Positive test**		33/48; 68.8%	3/8; 37.5%	36/56; 64.3%	5/37; 13.5%	1/40; 2.5%	1/6; 16.7%	0/6; 0%	7/89; 7.9%	<0.0001 ***	<0.0001 ***	<0.0001 ***
**OTHERS**												
**Facial vs. extra-facial vs.** **equivocal phenotype**		Facial 27/48; 56.2% Extra-facial 13/48; 27.1% Equivocal 8/48; 16.7%	Facial 2/8; 25% Extra-facial 5/8; 62.5% Equivocal 1/8; 12.5%	Facial 29/56; 51.8% Extra-facial 18/56; 32.1% Equivocal 9/56; 16.1%	Facial 17/37; 45.9% Extra-facial 13/37; 35.1% Equivocal 7/37; 18.9%	Facial 20/40; 50% Extra-facial 9/40; 22.5% Equivocal 11/40; 27.5%	Facial 3/6; 50% Extra-facial 2/6; 33.3% Equivocal 1/6; 16.7%	Facial 3/6; 50% Extra-facial 0/6; 0% Equivocal 3/6; 50%	Facial 43/89; 48.3% Extra-facial 24/89; 27% Equivocal 22/89; 24.7%	0.4703	0.8824	0.6151
**Annular globular pattern**		3/48; 6.2%	1/8; 12.5%	4/56; 7.1%	5/37; 13.5%	2/40; 5%	0/6; 0%	0/6; 0%	7/89; 7.9%	1	0.7307	0.2867
**Perifollicular linear** **projections/ pigmented circles with thick line(s) originating from them**		1/48; 2.1%	0/8; 0%	1/56; 1.8%	0/37; 0%	4/40; 10%	0/6; 0%	0/6; 0%	4/89; 4.5%	0.649	1	1
**Red circles**		5/48; 10.4%	1/8; 12.5%	6/56; 10.7%	0/37; 0%	0/40; 0%	1/6; 16.7%	0/6; 0%	1/89; 1.1%	0.0135 *	0.1183	0.0655
**Pseudonetwork**		15/48; 31.2%	1/8; 12.5%	16/56; 28.6%	11/37; 29.7%	17/40; 42.5%	2/6; 33.3%	3/6; 50%	33/89; 37.1%	0.3677	0.6761	1

* *p* < 0.05, ** *p* < 0.01, *** *p* < 0.001.

**Table 3:** Performance of selected melanoma screening algorithms.

Combined Performance on Training and Validation Sets									
	Sensitivity, %	Specificity, %	Compared to Our Model, *p*	Balanced Accuracy	PPV	NPV	Sensitivity, *p*	Specificity, *p*	Balanced Accuracy, *p*
**3-point checklist**	64.3%	92.1%	0.0046 **	78.2%	83.7%	80.4%	0.008 **	0.61	0.1407
**7-non melanoma features to rule out melanoma**	10.7%	84.6%	<0.0001 ***	47.5%	30%	60%	<0.0001 ***	0.5103	<0.0001 ***
**7-point checklist**	92.9%	58.4%	<0.0001 ***	75.6%	58.4%	92.9%	0.5254	<0.0001 ***	0.0007 ***
**‘Chaos and Clues’**	89.3%	75.3%	<0.0001 ***	82.3%	69.4%	91.8%	1	0.0318 *	0.1048
**CASH algorithm**	67.9%	93.3%	0.0148 *	74.9%	86.4%	82.2%	0.0233 *	0.4318	0.3119
**Menzies method**	67.9%	82%	0.499	74.9%	70.4%	80.2%	0.0233 *	0.2886	0.0136 *
**Proposed algorithm**	87.5%	88.8%	N/A	88.1%	83.1%	91.9%	N/A	N/A	N/A
**Performance on training set**									
	**Sensitivity, %**	**Specificity, %**	**Compared to our model,** ***p***	**Balanced** **accuracy**	**PPV**	**NPV**	**Sensitivity, *p***	**Specificity, *p***	**Balanced** **accuracy,** ***p***
**3-point checklist**	63.2%	95.2%	0.0218 *	79.2%	88.9%	81.1%	0.0682	0.489	0.4222
**7-non melanoma features to rule out melanoma**	10.5%	87.3%	0.0002 **	48.9%	33.3%	61.8%	<0.0001 ***	0.7768	<0.0001 ***
**7-point checklist**	97.4%	57.1%	<0.0001 ***	77.3%	57.8%	97.3%	0.1126	<0.0001 ***	0.0081 **
**‘Chaos and Clues’**	89.5%	77.8%	0.0339 *	83.6%	70.8%	92.5%	0.7344	0.0879	0.3225
**CASH algorithm**	79%	95.2%	0.3588	87.1%	90.9%	88.2%	0.7673	0.489	1
**Menzies method**	73.7%	84.1%	1	78.9%	73.7%	84.1%	0.3986	0.4222	0.1774
**Proposed algorithm**	84.2%	90.5%	N/A	87.3%	84.2%	90.5%	N/A	N/A	N/A
**Performance on validation set**									
	**Sensitivity, %**	**Specificity, %**	**Compared to our model,** ***p***	**Balanced** **accuracy**	**PPV**	**NPV**	**Sensitivity, *p***	**Specificity, *p***	**Balanced** **accuracy,** ***p***
**3-point checklist**	66.7%	84.6%	0.1824	75.6%	75%	78.6%	0.0921	1	0.2568
**7-non melanoma features to rule out melanoma**	11.1%	76.9%	0.0164 *	44%	25%	55.6%	<0.0001 ***	0.7249	0.0002 ***
**7-point checklist**	83.3%	61.5%	0.2888	72.4%	60%	84.2%	0.5959	0.118	0.0643
**‘Chaos and Clues’**	88.9%	69.2%	0.3711	79.1%	66.7%	90%	1	0.3234	0.2568
**CASH algorithm**	44.4%	88.5%	0.0162 *	66.5%	72.7%	69.7%	0.0038 **	1	0.0643
**Menzies method**	55.6%	76.9%	0.2278	79.1%	62.5%	71.4%	0.0209 *	0.7249	0.0382 *
**Proposed algorithm**	94.4%	84.6%	N/A	89.5%	81%	95.7%	N/A	N/A	N/A
**Performance on test set**									
	**Sensitivity, %**	**Specificity, %**	**Compared to our model,** ***p***	**Balanced** **accuracy**	**PPV**	**NPV**	**Sensitivity, *p***	**Specificity, *p***	**Balanced** **accuracy,** ***p***
**3-point checklist**	50%	86.7%	0.2207	68.3%	71.4%	72.2%	0.1432	1	0.2888
**7-non melanoma features to rule out melanoma**	20%	86.7%	0.0961	53.3%	50%	61.9%	0.007 **	1	0.0531
**7-point checklist**	90%	6.7%	0.0033 **	48.3%	39.1%	50%	1	<0.0001 ***	0.0012 **
**‘Chaos and Clues’**	100%	60%	0.0736	80.0%	62.5%	100%	1	0.2155	0.4616
**CASH algorithm**	60%	73.3%	1	66.7%	61.5%	83.3%	0.3017	0.6481	0.1721
**Menzies method**	80%	66.7%	0.6831	73.3%	60%	73.3%	1	0.388	0.2888
**Proposed algorithm**	90%	86.7%	N/A	88.3%	81.8%	92.9%	N/A	N/A	N/A

N/A—not applicable, PPV—positive predictive value, NPV—negative predictive value, * *p* < 0.05, ** *p* < 0.01, *** *p* < 0.001.

## Data Availability

The original contributions presented in this study are included in the article/Supplementary Material. Further inquiries can be directed to the corresponding author. The cases identified from the HAM10000 dataset were used as a test set to validate the proposed model: Tschandl, Philipp, 2018, “The HAM10000 dataset, a large collection of multi-source dermatoscopic images of common pigmented skin lesions”, https://doi.org/10.7910/DVN/DBW86T, Harvard Dataverse, V4. The file with the predictive model is openly available in Zenodo at http://doi.org/10.5281/zenodo.14533117.

## Supplementary Materials

The following supporting information can be downloaded at: https://www.mdpi.com/article/10.3390/cancers17040679/s1, Text S1: Dermoscopic analysis protocol; Text S2: Development of the predictive model, Figure S1: ROC curves of the predictive model.

## Abbreviations

The following abbreviations are used in this manuscript:

EEMLs	External ear melanocytic lesions
SSL	Special site location
EEMs	External ear melanomas
SAMPUS	Superficial Atypical Melanocytic Proliferations of Unknown Significance
MELTUMP	Melanocytic Tumours of Uncertain Malignant Potential
IAMPUS	Intraepidermal Atypical Melanocytic Proliferation of Uncertain Significance
N/A	Not applicable
SD	Standard deviation
PPV	Positive predictive value
NPV	Negative predictive value
LMM	Lentigo maligna melanoma
SSM	Superficial spreading melanoma
PLP	Perifollicular linear projections
