# Supplementary material for: Dermoscopy of External Ear Melanocytic Lesions: Performance of Selected Dermoscopic Screening Algorithms and Proposal of a New Predictive Model for Malignancy (AuriCheck Dermoscopic Algorithm)
Source: Cancers (Basel). 2025 Feb 17;17(4):679. doi: 10.3390/cancers17040679 (PMC11853154; doi:10.3390/cancers17040679)
Supplement: Supplementary file 1 [file cancers-17-00679-s001.zip › Supplementary Figure S1.pdf]

ROC curves of the predictive model

Training set

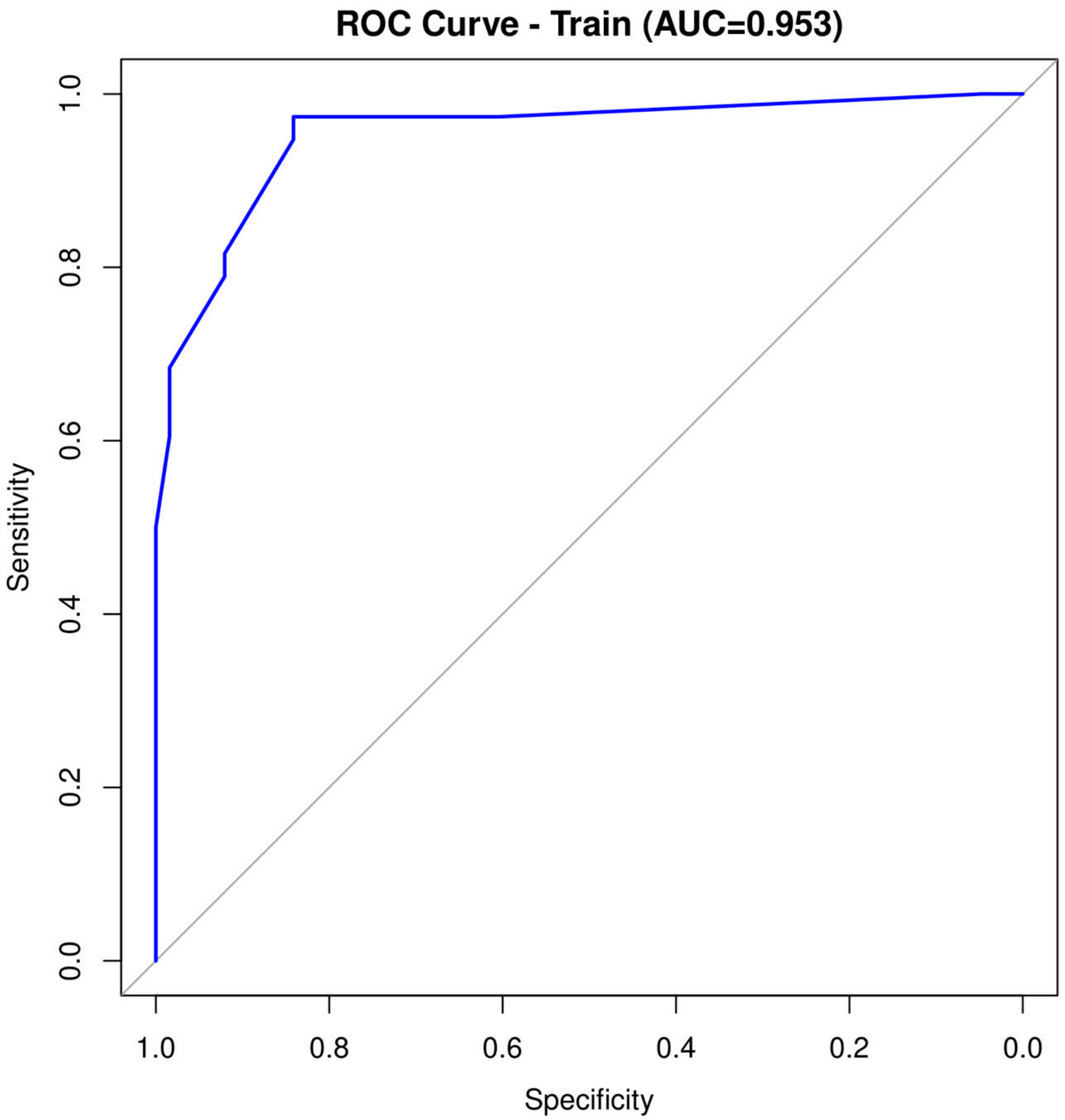

Validation set

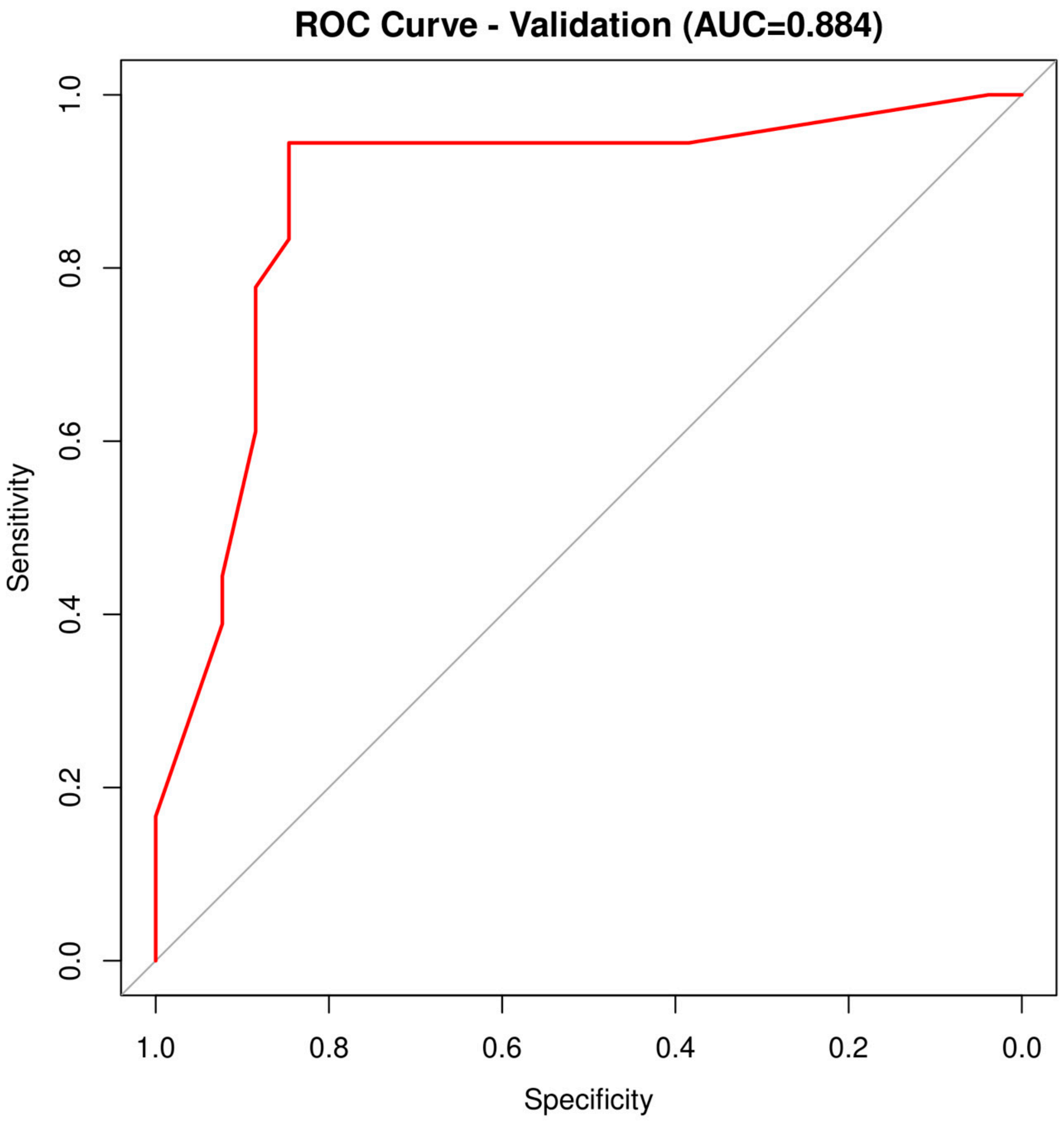

Test set – HAM10000

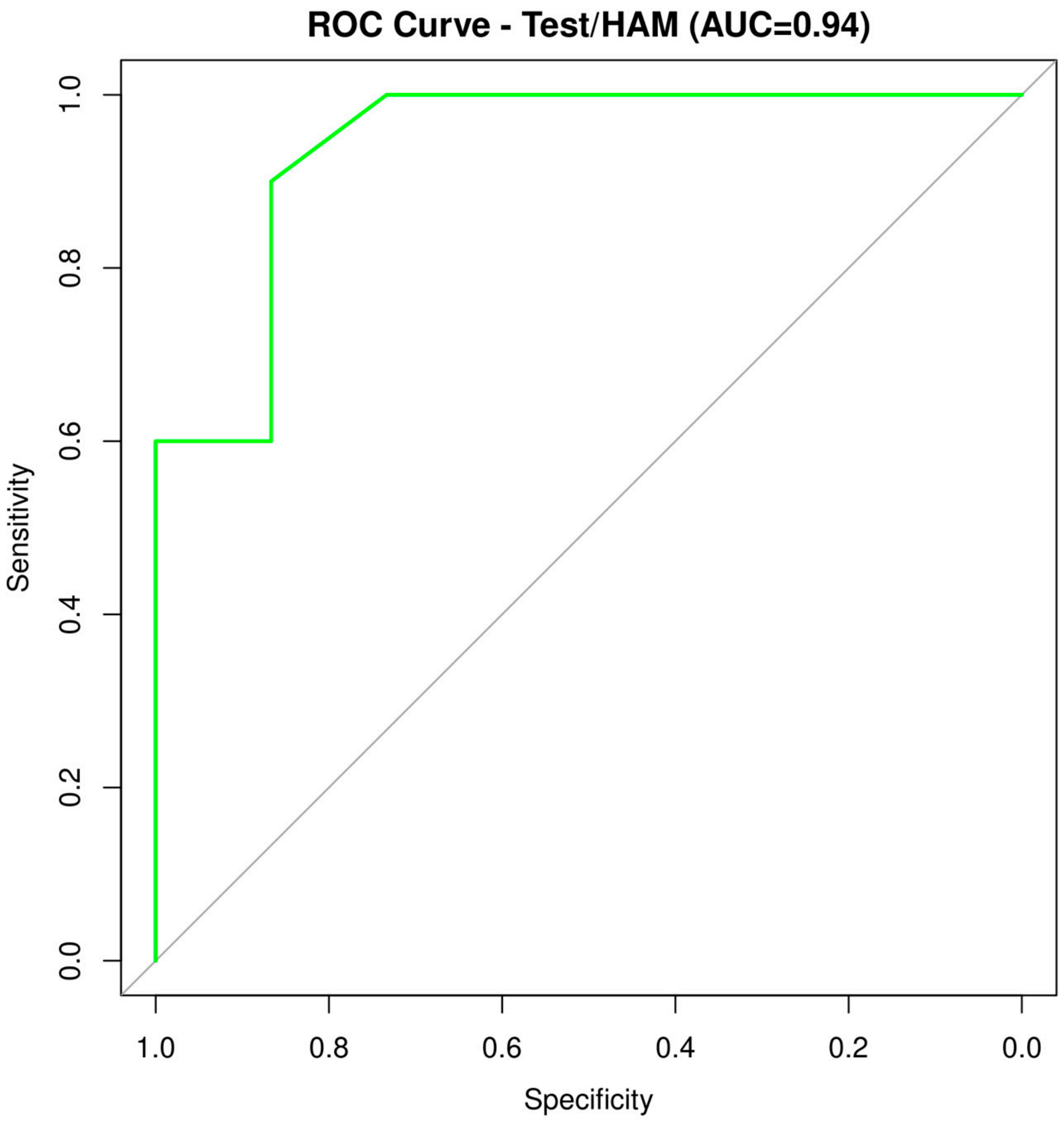

Training and validation sets combined

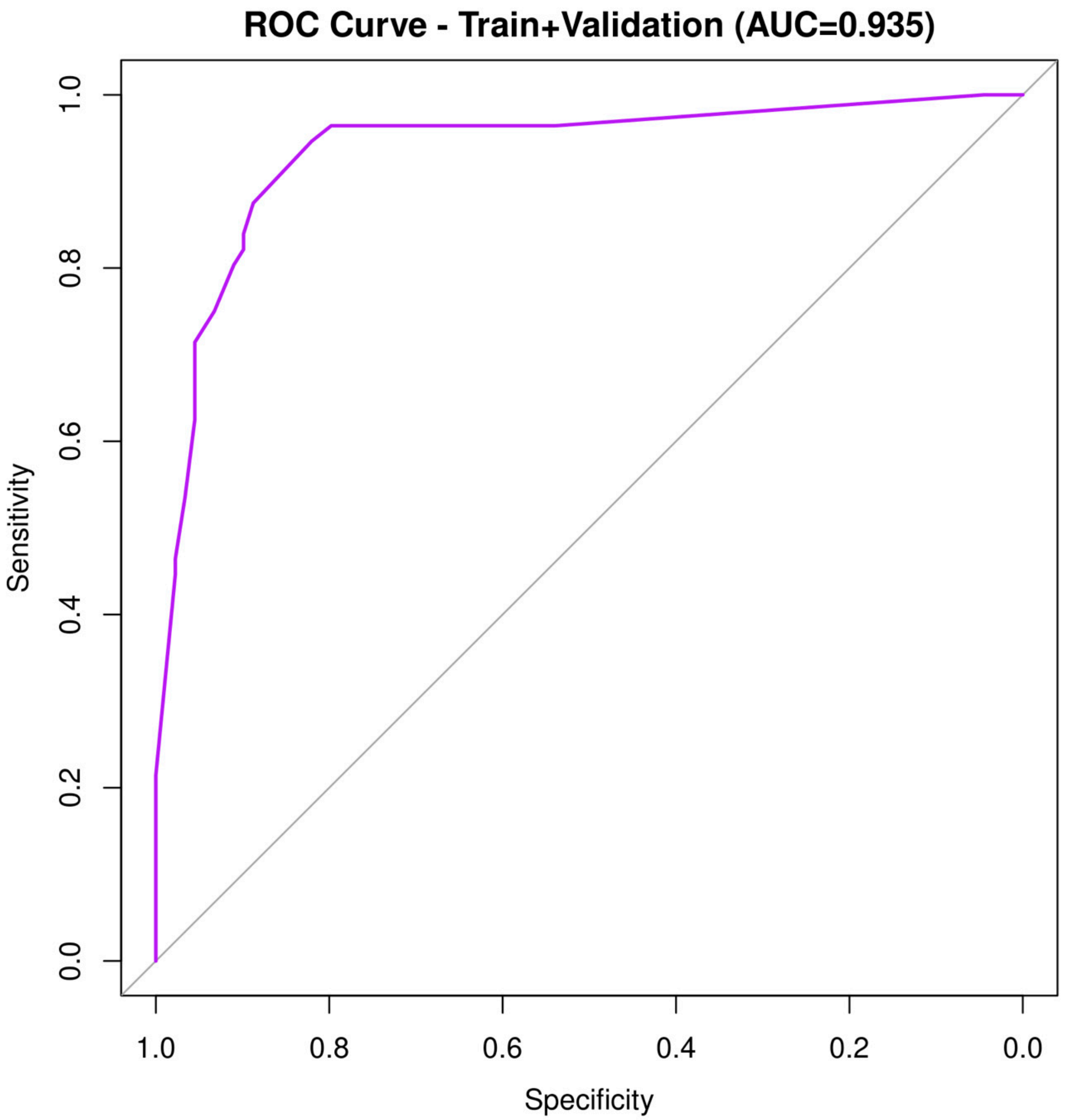

All sets combined

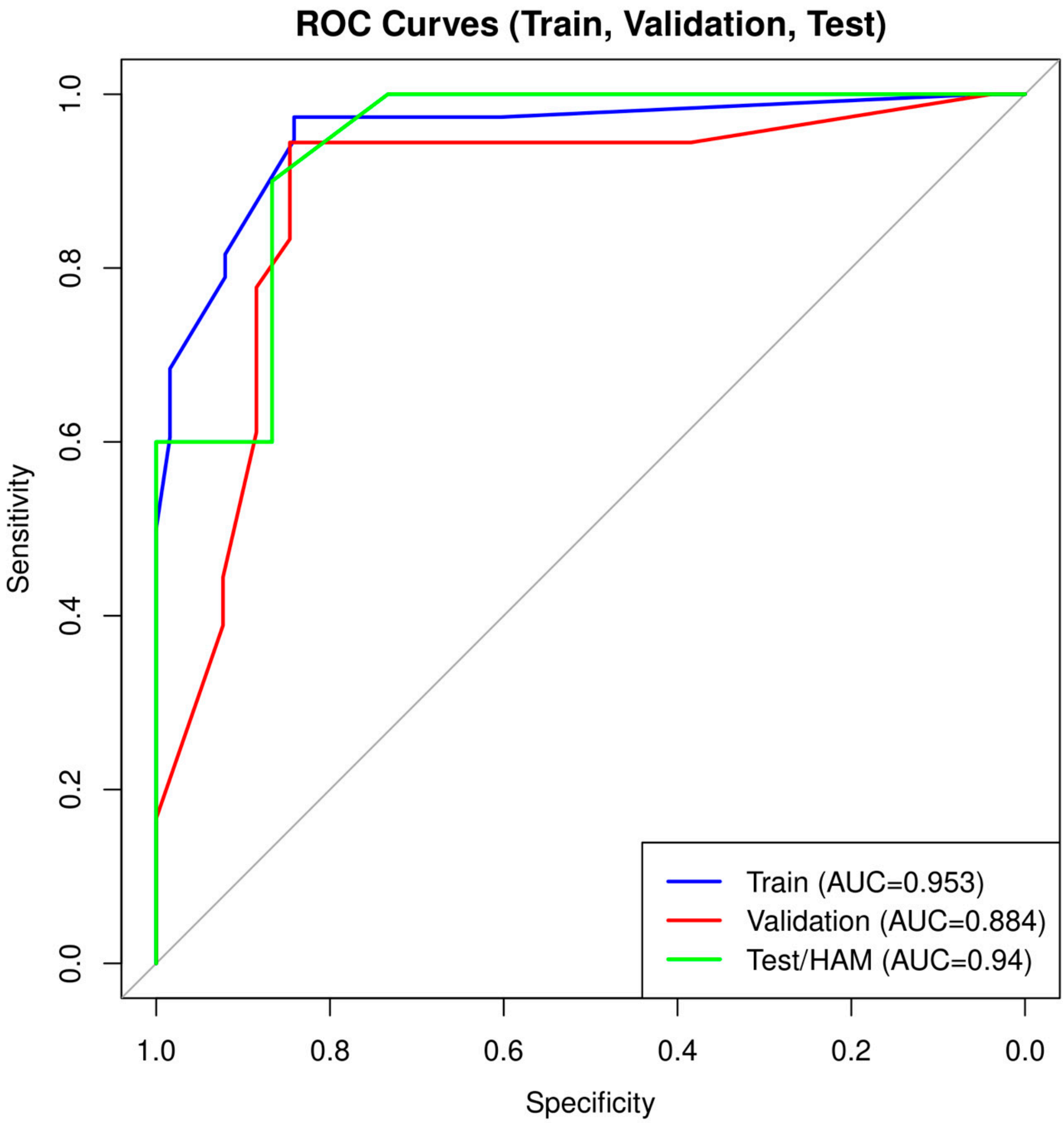

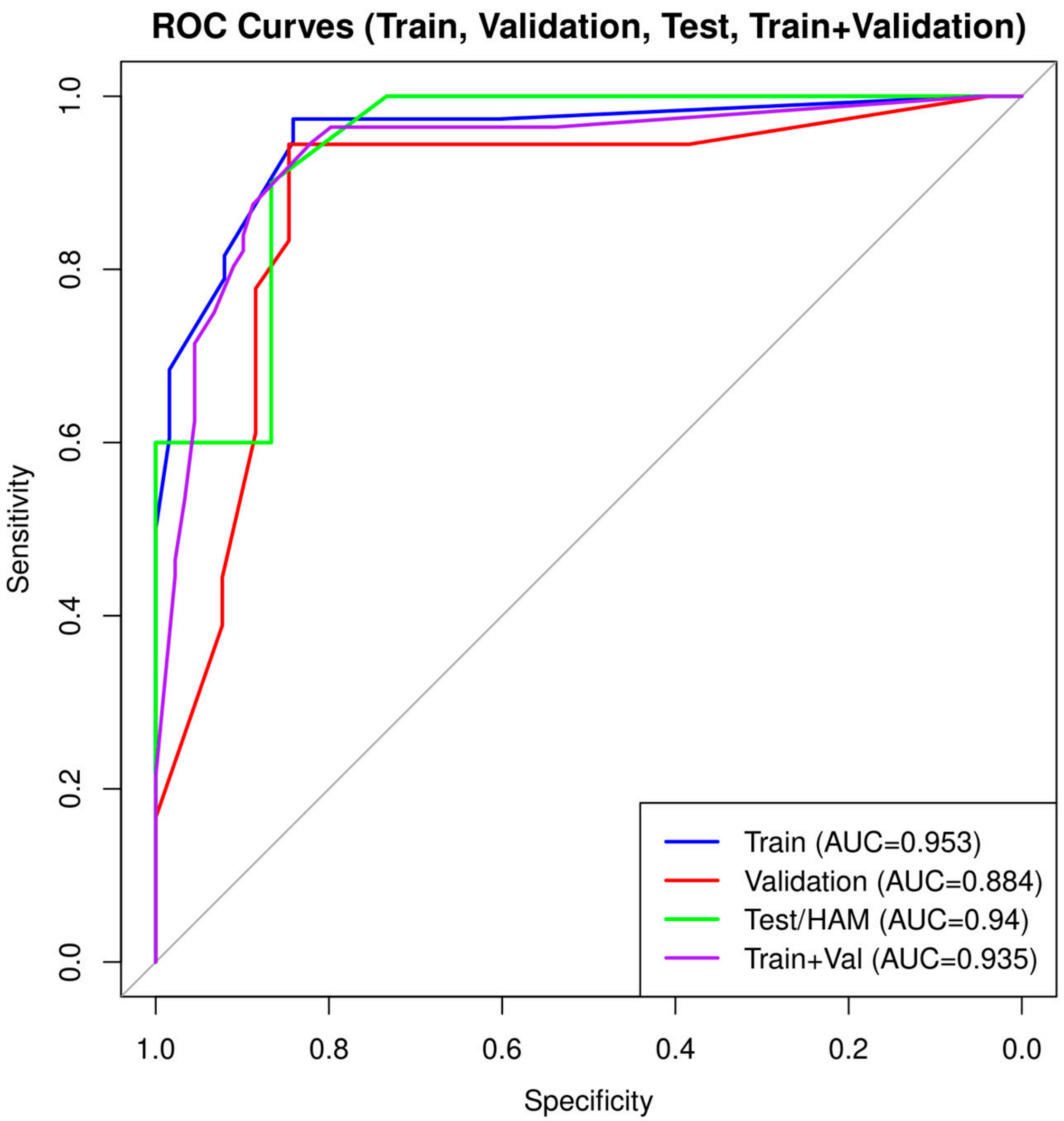

ROC Curves (Train, Validation, Test, Train+Validation)
Sensitivity
Specificity
1.0
0.8
0.6
0.4
0.2
0.0
Train (AUC=0.953)
Validation (AUC=0.884)
Test/HAM (AUC=0.94)
Train+Val (AUC=0.935)
