# Supplementary material for: Dermoscopy of External Ear Melanocytic Lesions: Performance of Selected Dermoscopic Screening Algorithms and Proposal of a New Predictive Model for Malignancy (AuriCheck Dermoscopic Algorithm)
Source: Cancers (Basel). 2025 Feb 17;17(4):679. doi: 10.3390/cancers17040679 (PMC11853154; doi:10.3390/cancers17040679)
Supplement: Supplementary file 1 [file cancers-17-00679-s001.zip › Supplementary Text S1.pdf]

Dermoscopic analysis approach

Dermoscopic images of each lesion were obtained using FotoFinder Vexia 800HD or 1000 videodermoscopes in non-polarized mode with 20x magnification [Medicam, Bad Birnbach, Germany], DermLiteCam dermoscopic camera [3Gen, San Juan Capistrano, USA], DermLite DL4 dermoscope [3Gen, San Juan Capistrano, USA] attached to an iPhone 11 [Apple, Cupertino, USA] or DermLite Foto dermoscope [3Gen, San Juan Capistrano, USA] attached to a Nikon Coolpix 995 digital camera [Nikon, Tokyo, Japan] – all in polarized mode with 10x magnification. Ultrasound gel was used as immersion if vascular structures were noted, otherwise isopropyl alcohol was utilized. No pressure was applied while acquiring images to facilitate visualisation of vascular structures.

Two independent investigators (J. Ż., M. Sł.), blinded to the diagnosis, evaluated the dermoscopic images using predefined criteria. To mitigate observation bias, videodermoscopic images were assessed by evaluators who were not involved in the sample collection. Interobserver agreement was measured using Cohen's kappa coefficient. If both evaluators did not reach a consensus, the evaluation by a third investigator (G.K.-W.) was considered decisive.

Statistical analysis

The distribution of quantitative variables was assessed using the Shapiro-Wilk test. Since all quantitative variables showed significant deviation from a normal distribution, the Wilcoxon test was employed to evaluate differences. Fisher exact test was used to evaluate binary variables. McNemar's test was used to compare the overall performance of the classifiers and proportion z tests were performed to compare sensitivities and specificities. A binomial test was conducted to evaluate gender proportions in the melanoma group. A p-value of < 0.05 was considered statistically significant. No data imputation was performed during the analysis.

Tschandl P, Rosendahl C, Kittler H. The HAM10000 dataset, a large collection of multi-source dermatoscopic images of common pigmented skin lesions. *Scientific Data.* 2018;5(1)**:**180161.
