# Supplementary material for: Dermoscopy of External Ear Melanocytic Lesions: Performance of Selected Dermoscopic Screening Algorithms and Proposal of a New Predictive Model for Malignancy (AuriCheck Dermoscopic Algorithm)
Source: Cancers (Basel). 2025 Feb 17;17(4):679. doi: 10.3390/cancers17040679 (PMC11853154; doi:10.3390/cancers17040679)
Supplement: Supplementary file 1 [file cancers-17-00679-s001.zip › Supplementary Text S2.pdf]

Development of the predictive model

The model file was deposited at:
https://zenodo.org/records/14533118?preview=1&token=eyJhbGciOiJIUzUxMiJ9.eyJpZCI6ImMyMjRmNDM3LWZkYzUtNDIzOC1iY2ZjLWZiMGRjNmU1N2QzNiIsImRhdGEiOnt9LCJyYW5kb20iOiI0NWZjMzBjYjk3YmUwODVhNWMzNDU0Zjc1NjQwMWI4OSJ9.d2aYwxllAQghANSbCYEYegSMEIVes0FkgksTos_51G5zgbJoFJy3ZvKxpSNOzU76Uh6TZS15IByLuImVk-UotQ
DOI: 10.5281/zenodo.14533117.

Example of R code to run the model is presented below:

```
#feature1 - Irregular pigmentation
#feature2 - Grey colour or blue colour
#feature3 - Irregular dots or globules
#feature4 - Hyperpigmented follicular openings or rhomboids
#feature5 - Red areas or polymorphous vessels

library(xgboost)
model <- xgb.load("model_final_features.model")
features <- c(
        as.numeric(input$feature1),
        as.numeric(input$feature2),
        as.numeric(input$feature3),
        as.numeric(input$feature4),
        as.numeric(input$feature5)
        )

        # Convert scaled features to a matrix with one row
        feature_matrix <- matrix(features, nrow = 1)
        # Predict using the XGBoost model
        prediction <- predict(model, feature_matrix)
        paste("Raw Prediction Score:", round(prediction, 4))

        # Determine the final decision based on the cutoff
        cutoff <- 0.4326725
        decision <- ifelse(prediction >= cutoff, "Positive", "Negative")
```
